# Clearance of erythrocytes from the subarachnoid space through cribriform plate lymphatics in female mice

**DOI:** 10.1016/j.ebiom.2024.105295

**Published:** 2024-08-22

**Authors:** Adrian Madarasz, Li Xin, Steven T. Proulx

**Affiliations:** Theodor Kocher Institute, University of Bern, Bern, Switzerland

**Keywords:** Subarachnoid haemorrhage, Red blood cells, Cranial nerves, Cribriform plate, Lymphatic vessels

## Abstract

**Background:**

Atraumatic subarachnoid haemorrhage (SAH) is associated with high morbidity and mortality. Proposed mechanisms for red blood cell (RBC) clearance from the subarachnoid space (SAS) are erythrolysis, erythrophagocytosis or through efflux along cerebrospinal fluid (CSF) drainage routes. We aimed to elucidate the mechanisms of RBC clearance from the SAS to identify targetable efflux pathways.

**Methods:**

Autologous fluorescently-labelled RBCs along with PEGylated 40 kDa near-infrared tracer (P40D800) were infused via the cisterna magna (i.c.m.) in female reporter mice for lymphatics or for resident phagocytes. Drainage pathways for RBCs to extracranial lymphatics were evaluated by *in vivo* and *in situ* near-infrared imaging and by immunofluorescent staining on decalcified cranial tissue or dural whole-mounts.

**Findings:**

RBCs drained to the deep cervical lymph nodes 15 min post i.c.m. infusion, showing similar dynamics as P40D800 tracer. Postmortem *in situ* imaging and histology showed perineural accumulations of RBCs around the optic and olfactory nerves. Numerous RBCs cleared through the lymphatics of the cribriform plate, whilst histology showed no relevant fast RBC clearance through dorsal dural lymphatics or by tissue-resident macrophage-mediated phagocytosis.

**Interpretation:**

This study provides evidence for rapid RBC drainage through the cribriform plate lymphatic vessels, whilst neither fast RBC clearance through dorsal dural lymphatics nor through spinal CSF efflux or phagocytosis was observed. Similar dynamics of P40D800 and RBCs imply open pathways for clearance that do not impose a barrier for RBCs. This finding suggests further evaluation of the cribriform plate lymphatic function and potential pharmacological targeting in models of SAH.

**Funding:**

10.13039/100000001Swiss National Science Foundation (310030_189226), SwissHeart (FF191155).

**Research in context**

**Evidence before this study**

The mechanisms for how red blood cells get cleared from the brain following subarachnoid haemorrhage are still unknown, however, a few studies showed evidence for clearance along cerebrospinal outflow pathways along cranial nerves, especially the olfactory and optic nerve, converging on cervical lymphatics. Recently, drainage through lymphatics at the dorsal dura has been proposed. Yet, the microanatomy of proposed pathways and the manner that red blood cells access draining lymphatic vessels at different anatomical locations have remained unclear.

**Added value of this study**

We have infused fluorescently-labelled washed red blood cells into the subarachnoid space in reporter mice for lymphatic vessels. By imaging different potential efflux pathways and the systemic vasculature following the injection as well as with the help of decalcification and histology we could find red blood cells draining through lymphatic vessels of the cribriform plate and in the lymphatics draining the eye to cervical lymphatics with similar dynamics as molecular tracer. We could not find relevant contribution for clearance through dorsal dural lymphatics, nor through phagocytosis by macrophages.

**Implications of all the available evidence**

This study has shown that cribriform plate lymphatics drain red blood cells from the subarachnoid space with similar dynamics as molecular tracer, implying direct open pathways that do not impose a barrier for particles of 6–7 μm size. Moreover, this anatomical tracer study identifies the major role of cribriform plate lymphatics for drainage of red blood cells from the subarachnoid space and suggests further investigation of this pathway and potential targeting in models of subarachnoid haemorrhage.

## Introduction

Atraumatic subarachnoid haemorrhage (SAH) is primarily caused by aneurysmal rupture^1^ and comprises 9.7% of all strokes.^2^ SAH occurs in a younger patient population than other stroke types and the high post-stroke morbidity and mortality lead to a significant burden of disease.^1^^,^^3^ Swift repair of the ruptured aneurysm through clipping or coiling is vital to control the haemorrhage^1^^,^^4^ but currently there are no validated therapies targeting the extravasated blood in the subarachnoid space (SAS).^5^ Emerging evidence shows that pathologic processes, described as early brain injury (EBI), commence promptly following haemorrhage,^5^^,^^6^ and are suggested to be the key prognostic factor for further complications.^5^ Blood extravasation into the SAS leads to an abrupt rise in intracranial pressure (ICP), triggering diminished cerebral perfusion and compromised vascular autoregulation.^7^ Moreover, deposition of blood in the SAS of patients suffering from SAH has been shown to disturb cerebrospinal fluid (CSF) circulation,^8^, ^9^, ^10^, ^11^ a finding that has been corroborated by murine studies.^12^^,^^13^ Lysis of red blood cells (RBCs) in the SAS and the consequent release of RBC breakdown products, especially free haemoglobin give rise to oxidation, inflammation, nitric oxide clearance, and oedema^14^ and contribute to EBI^15^^,^^16^ as well as delayed cerebral injury (DCI).^14^^,^^17^ Thus, impaired RBC and CSF clearance likely contribute to brain injury, as CSF circulation is indispensable for central nervous system (CNS) homeostasis.^18^, ^19^, ^20^

Despite increased recent attention, the pathways for CSF clearance remain a subject of ongoing discussion.^19^^,^^21^ CSF is produced by the choroid plexus in the brain ventricles and exits into the SAS through the foramina of the fourth ventricle.^22^ The SAS is delimited by the leptomeninges, comprised of the pia mater as the inner border and the arachnoid mater as the external border. The arachnoid mater expresses the adherens junction proteins vascular endothelial (VE)-cadherin^23^ and epithelial(E)-cadherin as well as the tight junction protein claudin-11^24^, ^25^, ^26^ and is impermeable to fluids, thus forming a blood-cerebrospinal fluid barrier.^26^, ^27^, ^28^ The dura mater, the outermost meningeal layer, contains a widespread network of blood vessels devoid of distinct barrier characteristics, and is host to lymphatic vessels.^18^^,^^29^ Cranial efflux of CSF has long been thought to occur through arachnoid villi directly into dural venous sinuses.^30^, ^31^, ^32^ However, historical and contemporary studies have shown CSF effluxes rather to lymphatic vessels. Studies from numerous mammalian species have demonstrated that CSF primarily reaches the lymphatics along perineural spaces of cranial nerves and then drains to the superficial (scLN) and deep (dcLN) cervical lymph nodes.^33^, ^34^, ^35^, ^36^, ^37^, ^38^, ^39^ However, with the recent rediscovery of dural lymphatics,^40^, ^41^, ^42^ it has also been suggested that CSF would drain through these vessels, overcoming the arachnoid barrier in a yet to be elucidated manner.^43^, ^44^, ^45^

Similar routes have been hypothesised for the egress of RBCs.^46^, ^47^, ^48^ Historical post-mortem investigations of the arachnoid villi in patients that had succumbed to SAH, have revealed an accumulation of RBCs as well as immune cell infiltration in the affected villi.^49^ Past research has also demonstrated that RBCs drain mainly through lymphatic pathways to cervical lymph nodes but were not able to describe the microanatomy of the pathway in detail.^48^^,^^50^ Studies in humans^50^^,^^51^ as well as in other mammalian species^47^^,^^52^ demonstrated accumulation of RBCs in perineural spaces of cranial nerves, especially those surrounding the optic nerve and olfactory nerve bundles. Our group has provided evidence of an interrupted arachnoid barrier as the underlying anatomical structure leading to patency of the cribriform plate pathway for particles up to at least 1 μm in diameter.^53^ Recent investigations using murine subarachnoid, intracerebral, or intraventricular haemorrhage models have suggested RBC clearance through dural lymphatic pathways.^54^, ^55^, ^56^ In addition, earlier investigations exploring RBC clearance from the SAS proposed erythrophagocytosis as a possible mechanism.^47^^,^^57^, ^58^, ^59^ In the brain, erythrophagocytosis and microglia activation in the SAS was reported after 24 h in subarachnoid and intracerebral haemorrhage models.^47^^,^^60^

In this study we performed infusions of autologous fluorescently labelled RBCs into the SAS via the cisterna magna (i.c.m.) in Prospero homeobox 1 (Prox1)-enhanced green fluorescent protein (EGFP)^61^ and C-X3-C motif chemokine receptor 1(CX3CR1)-GFP^62^ reporter mice for visualisation of RBCs in relation to lymphatic vessels and resident phagocytes, respectively. We investigated potential routes for RBCs to access extracranial lymphatics through *in vivo* intravital microscopy and *in situ* imaging performed immediately postmortem. Using confocal microscopy, we imaged decalcified cryosections of the cribriform plate and dorsal dural lymphatics as well as dural whole mounts. We demonstrate that labelled RBCs exhibit rapid drainage from the SAS to the lymphatic system and that the routes of egress are mainly associated with the cribriform plate rather than dural lymphatic vessels.

## Methods

### Ethics

All animal experiments were performed in accordance with Swiss law on animal welfare (Swiss Federal Welfare Act, TSchG) and were approved by the veterinary office of the canton of Bern, Switzerland (permit no. BE93/19 and BE141/2022). This study was performed in line with the ARRIVE guidelines for reporting animal research.

### Animal husbandry

Female Prox1-EGFP mice^61^ were bred in-house on the C57BL/6 J background. CCR2-RFP x CX3CR1-GFP mice^62^ were a kind gift from Dr. Israel F. Charo (UCSF, USA) and Dr. Richard Ransohoff (Boston, USA). CX3CR1-GFP mice were bred from the compound heterozygous mice. Female mice were utilised in similar tracer studies for CSF circulation in our lab before^38^^,^^39^ and male littermates are reserved for stroke model studies. Mice were kept in single ventilated cages under specific pathogen free conditions at a light–dark cycle of 13 h–11 h with food and water accessible *ad libitum*. Enrichment with spaces for retreat was provided. Experimental procedures were performed at an age of 8–14 weeks (total 48 animals).

### RBC collection and labelling

**Fig. 1:**
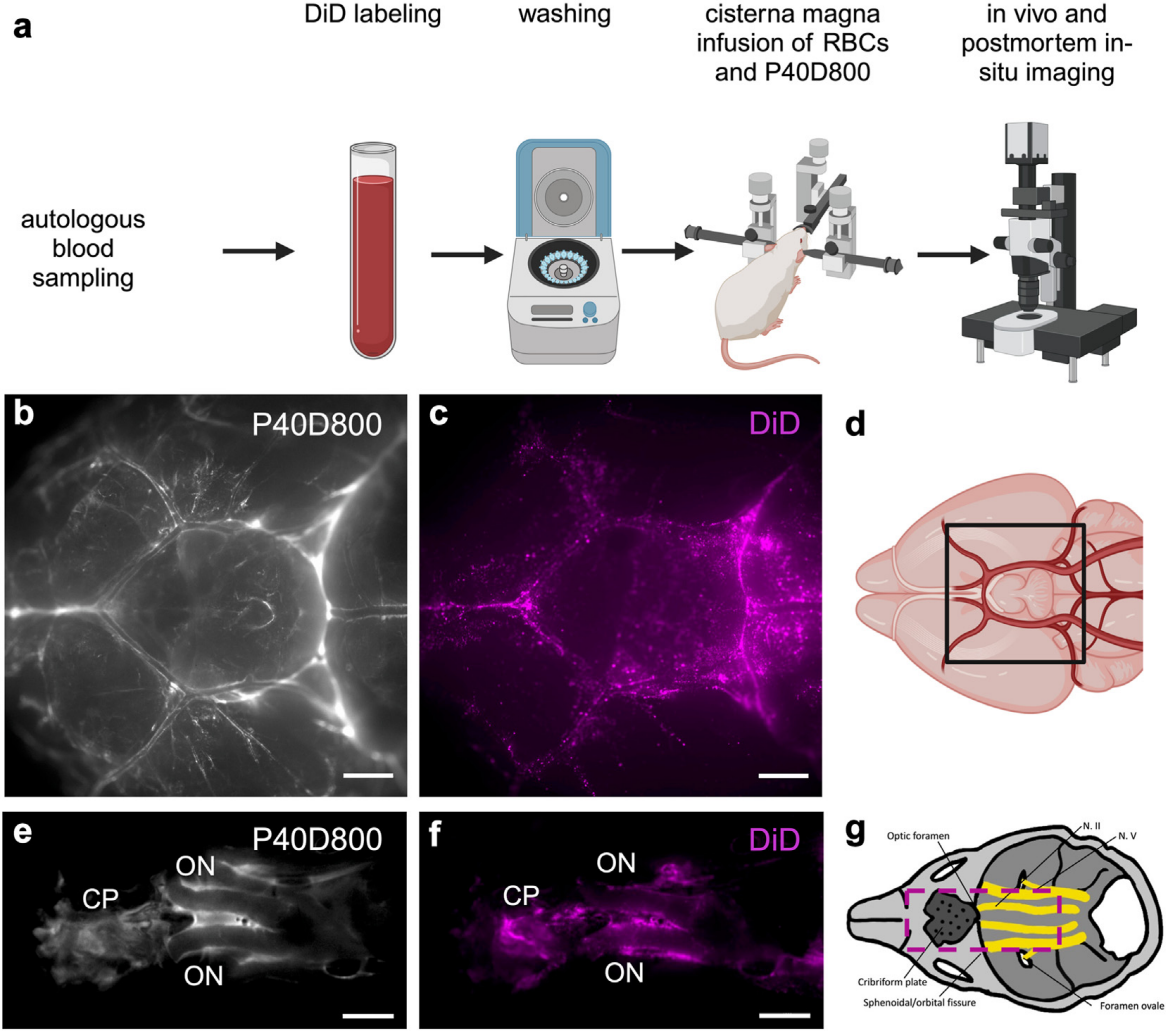
**Rapid tracer and RBC spread after i.c.m. infusion along ventral cisterns and perineural pathways**. a) Outline of the experimental procedure for co-infusion of DiD-labelled autologous RBCs (1.5 × 10^6^ RBCs in 1.5 μl) and P40D800 near-infrared tracer (1.5 μl) followed by *in vivo* and postmortem *in situ* imaging. b,c,e,f) Representative images of near-infrared tracer (white, b,e) and RBC (magenta, c,f) distribution directly post-mortem 15 min post-i.c.m. infusion (n = 8). b,c) Distribution along the ventral cisterns of the brain. d) Schematic illustration of the region imaged in b,c. e,f) Perineural spread of tracer and labelled RBCs at the skull base along ONs and at the cribriform plate. g) Schematic illustration of e,f. Scalebars: 1000 μm. a,d) created with bioRender. ON = optic nerve, CP = cribriform plate.

Under short isoflurane anaesthesia, mice were fixed in a restrainer, the saphenous vein was punctured with a lancet and 20 μl of blood were collected in a heparinised tube (Sarstedt, Nümbrecht, Germany). For labelling of RBCs for *in vivo* studies, blood was washed 3x in 200 μl phosphate-buffered saline (PBS, Gibco, Paisley, UK), centrifuged at 2000 g (7 min, 20 °C), and resuspended in 100 μl PBS for labelling with DiD (Invitrogen, Eugene, OR, USA) at a concentration of 0.005 mMol. RBCs were incubated on a shaker at 37 °C for 20 min. This was followed by washing 3x (200 μl PBS) and centrifuging at 240 g (6 min, 37 °C). PBS at 37 °C was used for washing and resuspending the RBCs after the incubation (Fig. 1a). For labelling of RBCs for decalcified tissue analysis, CellTracker™ deep red (CTdr, Invitrogen, Eugene, OR, USA) was used. For the labelling with CTdr, the RBCs were purified by Ficoll® (Cytiva, Uppsala, Sweden) gradient after the first washing step. After another wash, the RBCs were then suspended in 1.5 μl PBS with CTdr at a total concentration of 0.83 μMol for the labelling. RBCs were incubated on a shaker for 30 min at 37 °C and after washing (centrifuging at 340 g, 8 min, 37 °C) resuspended in 1.5 ml PBS and incubated for another 30 min. This was followed by the same washing and resuspension steps as described above. Finally, labelled cells were resuspended in 30 μl 1x PBS to reach the desired concentration for i.c.m. infusion.

### Flow cytometry analysis

Flow cytometry experiments were performed on an Attune NxT cytometer (Thermo Fisher Scientific, Reinach, Switzerland) and data was analysed using the FlowJo™ software (version 10, Ashland, OR, USA). The following gating strategy was applied to analyse the DiD or CTdr labelled subsets. RBCs were selected by size and granularity (FSC-A vs. SCC-A) and doublets excluded via a FSC-H and FSC-A density plot. Within the gated single RBCs the percentage of DiD or CTdr labelled RBCs was assessed by comparison to the fluorescence intensity of unlabelled RBCs that underwent the same procedures except for the labelling (Supplementary Fig. S1a-g).

### Infusion of RBCs into the tail vein

DiD labelled RBCs (35 × 10^6^, equivalent to 0.22% of RBCs in a 20 g mouse^63^) were suspended in 100 μl warm 0.9% saline (B. Braun, Sempach, Switzerland). Short anaesthesia was induced with 3.8% isoflurane mixed with >90% O_2_ in an induction chamber and maintained with a concentration of 1.8% applied by nose cone. The mouse was put on a heating pad and the tail additionally warmed with a warm water bag to increase venous distension. The tail was then disinfected with 70% ethanol and the RBC suspension injected into the lateral tail vein. Mice were left to recover under O_2_ supplementation and observed until showing regular breathing and normal awake behaviour.

### Infusion of RBCs and tracers i.c.m.

A single subcutaneous dose of 0.375 mg/kg buprenorphine (Temgesic, Eumedica Pharmaceuticals AG, Basel, Switzerland) was administered 30 min before the induction of anaesthesia to improve the anaesthetic effect during the following procedure. Anaesthesia with 80 mg/kg ketamine (Narketan, Vetoquinol AG, Bern, Switzerland) and 0.4 mg/kg medetomidine (Dormitor, Vetoquinol AG, Bern, Switzerland) was then injected intraperitoneally, followed by 1/3 of the initial dose after 30 min. On the absence of pain reflexes, the neck and cervical area was shaved as well as depilated and the mouse placed on a heating pad to maintain body temperature at 37 °C and the head fixed in a stereotaxic frame (RWD, Mainz, Germany). Surgical access to the cisterna magna was performed as previously described.^64^ In brief, the skin was incised at the midline over the occipital bone and cervical spinal cord and the underlying musculature was separated by blunt and for the innermost muscle layer by sharp dissection. Upon visualisation of the atlantooccipital membrane a bevelled glass capillary (Sutter instruments, Novato, CA, USA) with a diameter of 20–60 μm pulled on a Sutter P97 pipette puller (Sutter instruments Novato, CA, USA) mounted on a 10 μl gas tight syringe (Hamilton, Bonaduz, Switzerland) was slowly advanced to pierce the membrane at a perpendicular angle. After 2 min to allow for normalisation of ICP, 1.5 million RBCs suspended in 3 μl of 200 nM polyethylene glycol (PEG)ylated 40 kDa near-infrared tracer (P40D800)^65^ were infused with a syringe pump (Stoelting, Wood Dale, IL, USA) at a rate of 1 μl/min i.c.m. Similar infusion speeds of comparably low volumes were shown to not cause major interference with physiological CSF flow.^66^ After infusion, the capillary was glued to the surrounding tissue with tissue glue (Vetbond™, Fisher Scientific, Reinach, Switzerland) and was left in place for another 2 min to avoid backflow before being cut with scissors and sealed with a drop of glue. Following wound closure, mice were transferred to a monitoring station either for *in vivo* imaging or for monitoring until transcardial perfusion.

### *In vivo*, post-mortem *in situ* and *ex situ* imaging

Directly after i.c.m. infusion, mice were transferred to an epifluorescence microscope, placed on a heating pad to maintain body temperature at 37 °C, supplied with >90% oxygen and their temperature, heart rate and oxygenation were monitored with a Somnosuite® rodent anaesthesia machine (Kent Scientific, Torrington, CT, USA). Imaging was performed with a Zeiss AxioZoom.V16 microscope (Carl Zeiss, Feldbach, Switzerland), fitted with a Prime BSI Scientific sCMOS camera (Teledyne Photometrics, Tucson, AZ, USA), a light-emitting diode illumination system pE-4000 (CoolLED Ltd, Andover, UK), and ZEN 2.6 software (Carl Zeiss, Feldbach, Switzerland). The quality of infusion was assessed by dynamic *in vivo* through-skin NIR imaging of the scLNs, allowing for the visualisation of P40D800 tracer draining from the SAS. Upon reaching the predetermined time after infusion (15, 30, and 60 min) mice were euthanised by i.p. application of a ketamine/medetomidine overdose (660 mg/kg, 3.3 mg/kg). Imaging was performed directly after respiratory arrest. Skin was incised and dcLNs exposed. Images were acquired at 32x with an exposure of 50 ms for the green (EGFP), 500 ms for the far-red (DiD) and 100 ms for the NIR channel (P40D800). Lymphatic vessels draining the orbit were exposed, traced back to the exit point from the orbit and images were acquired with identical settings. Close-ups and epifluorescence z-stacks for extended depth-of-focus reconstructions were acquired with individual settings. After removal of the skull cap, images of the excised brain and skull base were acquired. The circle of Willis was imaged at 16x, with an exposure of 500 ms for the far-red (DiD) and 25 ms for the NIR channel (P40D800), and the skull base at 10x with the same exposure time.

### I.c.m. infusion of ovalbumin

I.c.m. infusion was performed as described above. A total of 3 μl of Alexa647-conjugated ovalbumin (OVA-AF647, Thermo Fisher Scientific, Waltham, MA, USA) at a concentration of 2 mg/ml was infused at a rate of 1 μl/min. Animals were sacrificed by transcardial perfusion 2 h post i.c.m. infusion.

### Decalcification of the skull and spinal column

Prox1-EGFP mice were investigated 30 min and CX3CR1-GFP mice 2 h after i.c.m. infusion, respectively. Under deep isoflurane anaesthesia and in the absence of pain reflexes, intracardiac perfusion was performed with ice-cold PBS followed by 4% paraformaldehyde (PFA, Merk Darmstadt, Germany) in PBS. Mice were decapitated and after removal of soft tissue and the lower jaw, skulls and spinal columns were harvested and post-fixed in 4% PFA for 24 h followed by decalcification in 14% Ethylenediaminetetraacetic acid (EDTA, Sigma–Aldrich, Steinheim, Germany) for 7 days, with EDTA being refreshed every second day. This was followed by cryoprotection in 30% sucrose (Merk, Darmstadt, Germany) for 3 days. Tissue was embedded in O.C.T. (Tissue-Tek®, Sakura Finetek, Umkirch, Germany) and frozen on dry ice and 2-Methylbutane (Sigma–Aldrich, Steinheim, Germany). Tissue was stored at −80 °C until further use. Coronal sections of 30 μm and sagittal sections of 50 μm were cut on a cryostat (CryoStar, NX50, Epredia, Cham, Switzerland).

### Immunofluorescence staining

Decalcified cryosections were used for immunofluorescence staining. After thawing at room temperature for 10 min, sections were hydrated with PBS for 3 × 5 min followed by permeabilisation of membranes with 0.1% Triton X-100 (Sigma–Aldrich, Steinheim, Germany) in PBS for 10 min. Blocking was achieved by adding 10% donkey serum for 1 h before overnight incubation at 4 °C in 10% donkey serum containing the primary antibody. Primary antibody was washed off in 2 washing steps with PBS and the secondary antibody was applied in 2% donkey serum and left for incubation for 2 h in the dark at room temperature. Then, slides were washed twice, stained for DAPI for 15 min, mounted on glass slides and cover-slipped with Mowiol (Sigma–Aldrich, Steinheim, Germany). Overview images were acquired on a Zeiss AxioZoom.V16 fluorescence microscope and high-resolution images also used for 3D reconstructions on a Zeiss LSM800 confocal microscope (Carl Zeiss, Feldbach, Switzerland). Further image analysis was performed with Fiji software^67^ or Imaris 9 (Oxford instruments, Abingdon, UK).

### Antibodies

Primary antibodies used in these experiments: rat anti-TER-119 1:100 dilution (BD Biosciences Cat# 553671, RRID:AB_394984), goat anti-E-cadherin 1:100 dilution (R&D Systems Cat# AF748, RRID:AB_355568). Secondary antibodies used in these experiments: donkey anti-rat Cy™3 1:300 dilution (Jackson ImmunoResearch Labs Cat# 712-165-150, RRID:AB_2340666), donkey anti-goat Cy™3 1:300 dilution (Jackson ImmunoResearch Labs Cat# 705-165-147, RRID:AB_2307351).

### Dural whole mounts

Dural whole mounts were dissected and prepared as described previously.^68^ In brief, Prox1-EGFP reporter mice were perfused transcardially with 10 ml ice cold PBS 30 min after i.c.m. infusion of CTdr-labelled RBCs. The calvaria was separated from the skull close to the skull base and carefully separated from the brain. After post-fixation in 2% PFA for 24 h, the dura mater was dissected from the calvaria under a stereomicroscope, mounted and carefully cover-slipped with Mowiol. Z-stacks from the confluence of sinus and the lateral borders of the transverse sinus were acquired on a LSM 800 confocal microscope.

### Quantification of RBCs in lymphatic vessels

Quantification was performed on 3D reconstructions of z-stack confocal images of decalcified coronal sections of the cribriform plate and sagittal sections of the cranium with dorsal dural lymphatics preserved as well as on dural whole mounts. Quantification was done with support of Imaris 9 to aid in the detection of CTdr labelled RBCs using the spot detection function. Automated counting of RBCs per field of view (FOV) was corrected manually when needed and association of the RBCs with Prox1-EGFP lymphatic vessels was assessed. Counting only RBCs outside of the arachnoid, be it in the subdural space at the dorsal side or directly at the cribriform plate, allowed for comparison of efflux pathways regardless of the cells in the SAS that were present in some of the images acquired. The percentage of RBCs in or on lymphatic vessels as a part of all the RBCs in the FOV was calculated. Due to the variability of lymphatic vessel size and number on the slides, we further normalised the percentage to the total area occupied by lymphatic vessels per FOV. The area was calculated on maximum intensity projections using uniform thresholds for decalcified cryosections and whole mounts, respectively. Due to the obvious differences in the anatomy of the tissues being quantified, blinding was not possible. For the cribriform plate and for dorsal dural lymphatics, 4 sections per animal from 4 separate animals were quantified. For dorsal dural lymphatics a total of 4 locations (2 from the confluence of sinus as well as 1 from each lateral side of the transverse sinus) were quantified in 4 separate whole mounts.

### Quantification of phagocytosis in CX3CR1-GFP reporter mice

Phagocytes (CX3CR1-GFP) and RBC-CTdr (Cy5 channel) positive area was quantified on confocal images of coronal sections of decalcified cranium. Images were acquired at the level of the olfactory bulbs directly above the cribriform plate. GFP^+^ and CTdr^+^ area was measured in semi-automated fashion with uniform thresholds set for both channels respectively and manual exclusion of the bone of the cribriform plate, if present. Three slides per mouse from a total of 4 mice were analysed. The area that is both CTdr and GFP positive was calculated as a percentage of the total GFP positive area. This allowed for quantification of CTdr labelled RBC phagocytosis even though a limited Cy5 autofluorescence is present in phagocytes due their organelles. With the area being calculated by thresholds set in advance, bias could be evaded without the need for blinding.

### Statistics

Statistical analyses were performed with GraphPad Prism 9 (La Jolla, CA, USA). P-values less than 0.05 were deemed as statistically significant. Data is presented as mean ± SD. Data was tested for normal distribution using the Shapiro–Wilk test. Groups of data with non-normal distribution were compared with the Mann-Whitney-test (two-tailed) or the Kruskal-Wallis-test with Dunn's multiple comparisons.

### Role of funders

The funders were not involved in the study's design, data collection, analysis, interpretation or reporting.

## Results

### RBCs rapidly distribute along ventral SAS cisterns and cranial nerves after i.c.m. infusion

**Fig. 3:**
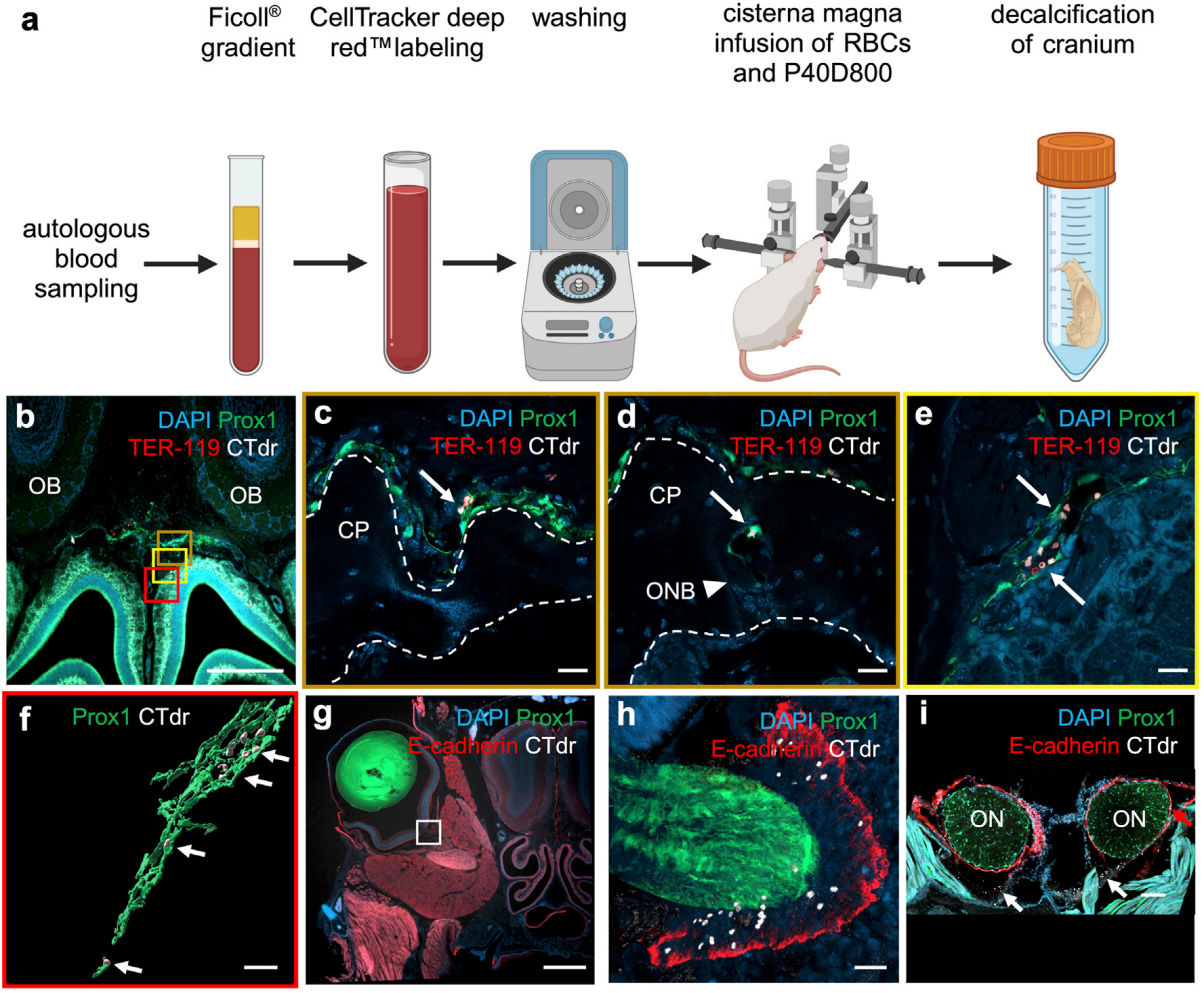
**RBCs clear along olfactory and optic nerve pathways**. a) Outline of experimental procedure for co-infusion of CTdr-labelled autologous RBCs (1.5 × 10^6^ RBCs in 1.5 μl) and P40D800 (1.5 μl) tracer into the cisterna magna followed by cardiac perfusion, skull harvest and decalcification. b,c,d,e) Representative coronal images of decalcified tissue at the cribriform plate region 30 min post-i.c.m. infusion (n = 4). b) Overview of cribriform plate region. c,d) Magnification of b (brown square) at different levels of z-stack, showing RBC efflux over the cribriform plate in one continuous lymphatic vessel imaged at different z-levels. Labelled RBCs (arrows, white = labelling, red = staining) are visible within the lumen of a lymphatic vessel above the cribriform plate (c) and within the lymphatic vessel crossing the bone of the cribriform plate (d). e) Magnification of b (yellow square). Labelled RBCs (arrows) draining within the lymphatics of the nasal mucosa. f) Magnification of b (red square). 3D reconstruction of labelled RBCs in the lumen of a lymphatic vessel in the nasal mucosa. g) Representative coronal images of decalcified tissue of the ON 30 min post-i.c.m. infusion (n = 4). h) Magnification of g (white square). Labelled RBCs (white) visible in the extension of the SAS around the ON delineated by the E-cadherin-stained arachnoid (red). i) Representative coronal maximum intensity projection of decalcified tissue showing the continuous E-cadherin-stained arachnoid barrier layer ensheathing the ON at the level of the cavernous sinus. White arrows = RBCs outside of arachnoid barrier, red arrow = subarachnoid RBCs a) Created with Biorender. Scalebars: b 500 μm; c,d,e,f 20 μm; g 1000 μm; h 30 μm; i 100 μm. CP = cribriform plate, ONB = olfactory nerve bundle, ON = optic nerve.

Previous studies from our group have shown that, following intraventricular (i.c.v.) infusion, macromolecular tracers distribute along the ventral cisterns of the brain and clear through perineural routes, including the spaces along the optic nerves crossing into the orbit as well as those surrounding the olfactory nerve bundles passing the cribriform plate.^38^^,^^39^^,^^69^ Thus, we first assessed whether labelled RBCs spread in the SAS and can access the same efflux pathways following i.c.m. infusion. For visualisation of RBCs we established two cell labelling techniques depending on the experiment: a DiD cell membrane labelling protocol with superior brightness for *in vivo* or *in situ* detection (Fig. 1a, Supplementary Fig. S1a–d, Supplementary Video S1) and a CTdr labelling that is retained intracellularly during decalcification and staining procedures (Fig. 3a, Supplementary Fig. S1e–g).

To compare the distribution and dynamics of labelled RBCs with P40D800 we co-infused both i.c.m. of Prox1-EGFP mice (Fig. 1a). *Ex vivo* epifluorescence imaging of the circle of Willis 15 min after i.c.m. infusion revealed RBCs spreading to the rostral part of the ventral cisterns as well as along the perivascular space of the medial cerebral artery, identical to the localisation of P40D800 (Fig. 1b, c, d). Thus, CSF circulation pathways along the ventral SAS in mice showed no size restriction for RBCs. Furthermore, both RBCs and tracer accumulated within the perineural spaces of the optic nerves and also reached the region of the cribriform plate, the latter known to be a major CSF efflux pathway in mice (Fig. 1e, f, g).^53^^,^^70^ The presence of RBCs in all regions of the ventral SAS already 15 min post-i.c.m. infusion indicates dynamics comparable to macromolecular tracer. Control experiments with mice infused with unlabelled RBCs, along with P40D800 to confirm successful infusion, did not demonstrate any signal above threshold in the imaged regions (Supplementary Fig. S2).

Previous studies have demonstrated spread of tracers along the spinal SAS after injection into the cranial CSF spaces, with the major lymphatic efflux pathway identified in the lumbosacral area.^71^ To account for this additional potential efflux route, we investigated the distribution of labelled RBCs on decalcified spinal tissue. While 30 min following i.c.m. infusion sparse accumulations of CTdr labelled RBCs could be detected on the cervical and thoracic level, only occasional single RBCs were present at the lumbar and sacral levels (Supplementary Fig. S3). These findings reveal similar dynamics as seen for the spread of molecular tracer along the spinal cord after i.c.v. infusion in mice.^71^

### RBCs drain from the SAS to cervical lymphatics within 15 min

**Fig. 2:**
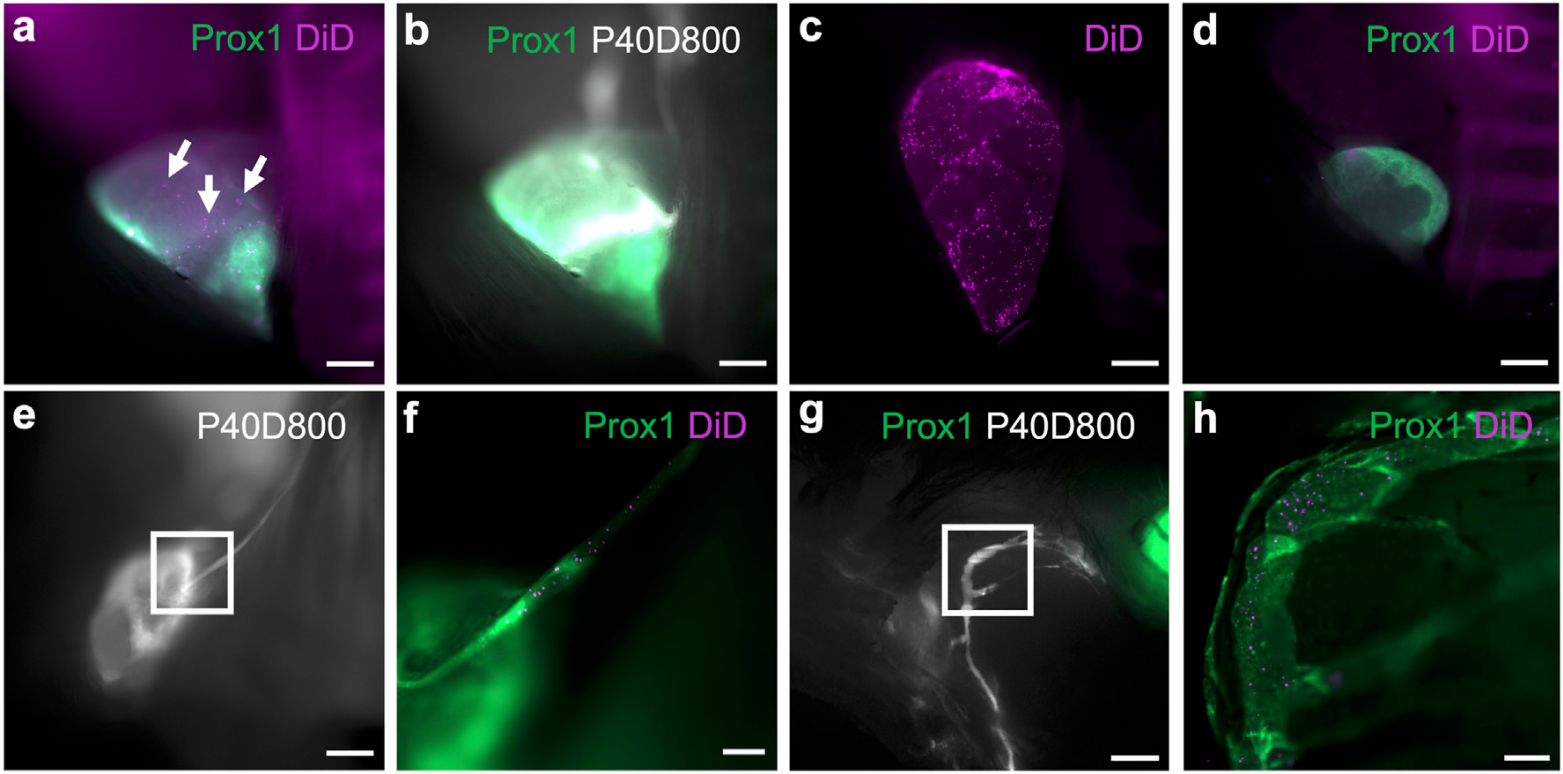
**Labelled RBCs and macromolecular tracer drain from the SAS through lymphatic vessels and arrive in the dcLNs by 15 min**. a,b) Representative *in situ* post-mortem images of dcLN 15 min post-i.c.m. infusion of DiD-labelled autologous RBCs showing labelled RBCs (pink, arrows, a) and P40D800 tracer (white, b) arriving simultaneously (n = 8). c) Representative extended depth of focus image of dcLN 15 min post-i.c.m. infusion showing accumulation of labelled RBCs. d) Control image of dcLN 15 min post-i.c.m. infusion of unlabelled RBCs (n = 3). e) Representative *in situ* post-mortem image showing drainage of P40D800 along the afferent lymphatic vessels to the dcLN 30 min post-i.c.m. infusion (n = 7). f) Magnification of e (white square), acquired with different channels. Representative extended depth of focus image for the drainage of labelled RBCs within the afferent lymphatic vessel of the dcLN 30 min post-i.c.m. infusion. g) Representative *in situ* post-mortem image for the lymphatic drainage of P40D800 from the orbit 30 min post-i.c.m. infusion, overlying skin was removed (n = 7). h) Magnification of g (white square), acquired with different channels. Representative extended depth of focus image showing lymphatic drainage of labelled RBCs from the orbit 30 min post-i.c.m. infusion. Scalebars: a,b,c,d,g 500 μm; e 1000 μm; f,h 200 μm.

As shown in previous tracer experiments from our group and others, lymphatic efflux to the cervical lymph nodes provides a major drainage pathway for CSF.^38^^,^^39^^,^^70^^,^^71^ As RBCs were visible along perineural clearance pathways, we hypothesised that RBCs could drain to cervical lymphatics following i.c.m. infusion. Thus, we investigated the presence of DiD labelled RBCs in the scLNs and dcLNs directly postmortem after euthanasia at 15, 30 and 60 min (n = 7–8 mice per timepoint) post i.c.m. infusion. Surprisingly, RBCs could be detected in dcLNs already 15 min post i.c.m. infusion, coinciding with signal enhancement from the P40D800 tracer (Fig. 2a, b, c). In control mice infused with unlabelled RBCs no signal above background was visible at the settings used to detect DiD (Fig. 2d). RBC drainage to scLNs was slightly delayed as compared to dcLNs, with only sparse individual cells detected after 15 min and groups of cells visible at 30 min post i.c.m. infusion. Continuous drainage of RBCs and tracer through Prox1-EGFP^+^ afferent cervical lymphatics to the dcLNs was observed at later timepoints (Fig. 2e, f, Supplementary Fig. S4). The similar efflux dynamics between P40D800 tracer and RBCs imply that RBCs are drained into the dcLNs through the bulk flow of CSF from the SAS.

As we could detect RBCs in the scLNs, which are known to receive lymph from lymphatic vessels draining tissue surrounding the mouse eye,^38^ we exposed and imaged the Prox1-EGFP^+^ lymphatic vessels draining the orbital cavity directly *in situ* postmortem 15, 30 and 60 min post i.c.m. infusion. We could observe P40D800 tracer as well as RBCs in lymphatics draining the orbit (Fig. 2 g, h). However, the dynamics were more variable compared to the dcLNs. Whilst all 7 mice assessed after 15 min showed orbital tracer efflux, RBCs were visible in 6 mice. Numerous RBCs could be observed in these lymphatic vessels in 4 mice, while sparse RBCs detected in 2 mice. Investigations of later timepoints confirmed this pathway to be accessible for RBCs in the majority of mice (6 out of 8 at 30 min, and 7 out of 8 at 60 min).

In summary, our data shows that both RBCs and macromolecular tracers are draining from the SAS through cervical lymphatic vessels converging on scLNs and dcLNs. These findings together prompted us to undertake a detailed anatomical investigation of potential efflux sites such as the cribriform plate, the optic nerve and dorsal dural lymphatics.

### Clearance of RBCs from the SAS through lymphatics at the cribriform plate

The cribriform plate has been established as one of the major contributors to CSF efflux to the dcLNs, with the pathways to lymphatics open to large particles such as 1 μm diameter PEGylated beads.^19^^,^^53^^,^^72^ Thus, we aimed to investigate whether RBCs can also gain access to the lymphatics surrounding olfactory nerve bundles at the cribriform plate.

To explore efflux pathways on decalcified tissue sections, CTdr was used to label the RBCs before infusion. Mice were sacrificed and perfused 30 min post i.c.m. infusion and the craniums harvested for decalcification (Fig. 3a). Coronal sections of the cribriform plate region were cryosectioned and stained for TER-119 (isotype control stainings in Supplementary Fig. S5) to visualise the RBC membrane in addition to the CTdr labelling. Confocal imaging of coronal decalcified sections demonstrated that RBCs had crossed the cribriform plate within Prox1-EGFP^+^ lymphatic vessels (Fig. 3b–e). RBCs were found within the lymphatics directly above the cribriform plate (Fig. 3b and c) and within the lymphatic vessels associated with olfactory nerve bundles (Fig. 3d, Supplementary Video S2). Further imaging and 3D reconstructions confirmed RBCs draining within the lymphatics below the cribriform plate (Fig. 3e and f, Supplementary Video S3). Qualitative assessment from a total of 8 mice at 30 min post i.c.m. infusion revealed that RBCs were consistently found within lymphatic vessels along the olfactory nerve bundles and rarely could be found in the tissue spaces around the nerves. Our findings confirm that the pathways from the SAS to lymphatics at the cribriform plate are open to egress of murine RBCs.

### RBCs reach the SAS surrounding the optic nerve

*In situ* postmortem imaging indicated a potential RBC efflux along the SAS extending around the optic nerve. Furthermore, RBC efflux to lymphatics draining the orbit was observed (Fig. 2). Although small particles have been found outside the arachnoid barrier at the optic nerve's termination into the eye,^73^ it remains to be elucidated if RBCs can exit from the SAS along the optic nerve. To investigate this, we performed confocal imaging on coronal cryosections from animals perfused 30 min post i.c.m. infusion. Sections showing the most distal part of the optic nerve were selected and stained for E-cadherin to visualise the arachnoid barrier^26^ (Fig. 3g and h). Confocal imaging revealed that RBCs could reach the most distal part of the SAS surrounding the optic nerve (Fig. 3h). Surprisingly, despite our evidence of RBC drainage through orbit-draining lymphatics, on tissue sections we could not localise RBCs in the periorbital tissue. The cells remained confined within the perineural sheath delimitated by the arachnoid barrier, which appeared as a continuous E-Cadherin^+^ layer visualised by immunofluorescence staining (Fig. 3h, isotype control stainings in Supplementary Fig. S5). At more proximal parts of the optic nerve within the skull, we also confirmed an intact arachnoid barrier along the nerve with RBCs present in the SAS. However, at the level of the cavernous sinus we were able to visualise RBCs outside of the arachnoid barrier without being able to detect any discontinuity of the barrier itself (Fig. 3i). Our data shows that whilst the arachnoid, in line with previous reports,^74^ seemed to be continuous along the length of the optic nerve, RBCs could still be found outside of the E-cadherin positive barrier layer. However, the route to the lymphatics draining the orbit still remains to be elucidated.

### Minor role of dorsal dural lymphatics in RBC efflux

**Fig. 4:**
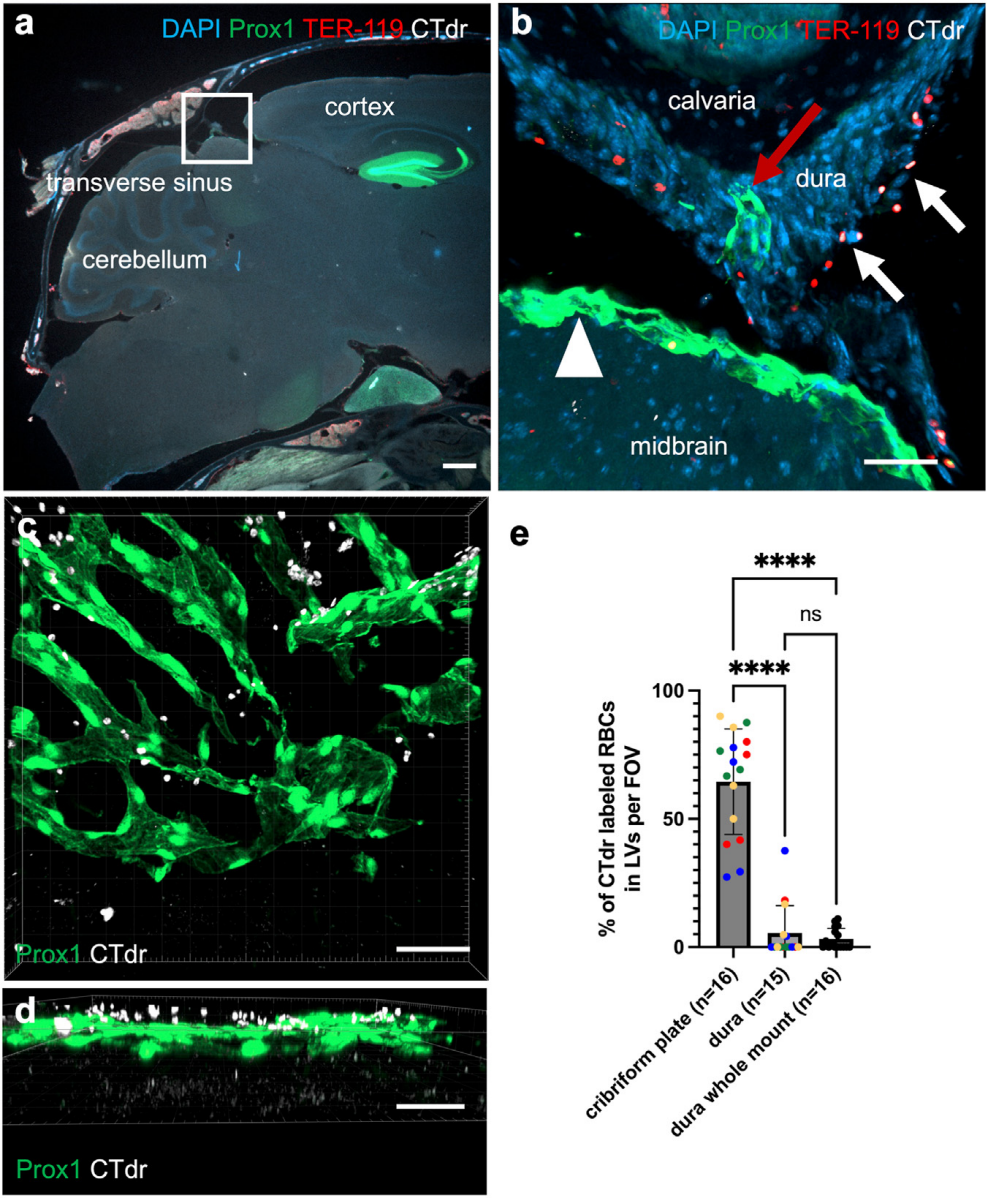
**Limited efflux of RBCs apparent through dorsal dural lymphatics**. a) Representative sagittal images of decalcified tissue at the transverse sinus region 30 min post-i.c.m. infusion of CTdr-labelled autologous RBCs (according to Fig. 3a). Square indicates where the close-up z-stacks of the transverse sinus close to the confluence of sinus were acquired. b) Representative sagittal 3D reconstruction of dorsal dural lymphatics from z-stacks acquired on decalcified tissue, 30 min post-i.c.m infusion (n = 4). Imaging close to the confluence of sinus. Single RBCs (white arrows, white = labelling, red = staining) are visible adjacent to dorsal dural lymphatics (red arrow) in the dural interstitium but not in the lumen of the lymphatic vessel (arrowhead = arachnoid). c) Representative 3D reconstruction of dorsal dural lymphatics (lateral transverse sinus) of a dural whole mount preparation harvested 30 min-post i.c.m. infusion (n = 4). *En face* view. d) Same 3D reconstruction as in c, lateral view. Labelled RBCs are outside of the lymphatic vessels. e) Cribriform plate lymphatics drain significantly more RBCs than dorsal dural lymphatics. Shown is the percentage of labelled RBCs inside the lymphatic vessels with 100% representing all labelled RBCs outside the arachnoid in the FOV. Same colour dots mark data points acquired from one animal (4 animals per group). ∗∗∗∗ = p < 0.0001 (Kruskal–Wallis test with Dunn's correction). Data presented as mean ± SD. Scalebars: a 500 μm; b,c,d 50 μm.

As recent publications have proposed immune cell and RBC efflux through dorsal dural lymphatics,^54^^,^^55^^,^^75^ we investigated the presence of RBCs within Prox1-EGFP^+^ lymphatic vessels 30 min after i.c.m. infusion on both cryosections (Fig. 4a and b) and dural whole mounts (Fig. 4c and d). Sagittal cryosections of the transverse sinus, close to the confluence of sinus showed that labelled RBCs are found in a (sub)dural space on the dorsal side of the skull (Fig. 4b), outside of the arachnoid as visualised by the Prox1^+^ reporter layer that is found associated with the E-cadherin^+^ barrier layer^23^^,^^26^ (Supplementary Fig. S6). The majority of labelled RBCs were localised in the dural interstitial space, without being directly associated with the dorsal dural lymphatics (Fig. 4b). These images suggest that RBCs can reach the dura in the region of the transverse sinus. Moreover, the RBCs do not appear to localise to the lymphatic vessels at this location.

To further corroborate our observations, we acquired confocal z-stacks of dural whole mount tissue harvested 30 min after i.c.m. infusion. Whilst on sagittal sections the dural lymphatics might only cover a small area, we were able to image a considerable amount of the dural lymphatics using whole mounts. In an *en face* projection, RBCs appeared to be associated with lymphatics (Fig. 4c). However, a lateral projection of the 3D reconstructions revealed that the majority of labelled RBCs were clearly outside the lymphatics (Fig. 4d, Supplementary Video S4). To compare the efflux of RBCs 30 min post i.c.m. infusion to dorsal dural lymphatics and through the cribriform plate, the percentage of labelled RBCs in or in direct contact with lymphatic vessels was calculated. Quantification (Fig. 4e) demonstrated that a significantly larger percentage of RBCs were associated with the lymphatic vessels of the cribriform plate (64.49 ± 20.55%) than with the dorsal dural lymphatic vessels on cryosections (5.42 ± 10.74%) as well as dural whole mounts (3.23 ± 4.08%, p < 0.0001, Kruskal–Wallis test with Dunn's correction). Given the different sizes of lymphatic vessels in the images and to provide a more unbiased approach, we normalised to the area occupied by lymphatics in each FOV (Supplementary Fig. S7). With this approach, the outcome remained unchanged, with significantly more RBCs associated with the lymphatics of the cribriform plate (22.27 ± 20.55%) compared to dural lymphatics (dorsal dural lymphatics 0.90 ± 1.94%, dural whole mounts 0.60 ± 1.18%, p < 0.0001, Kruskal–Wallis test with Dunn's correction).

### Retention of RBCs in cervical lymph nodes but no evidence of phagocytosis at the cribriform plate

**Fig. 5:**
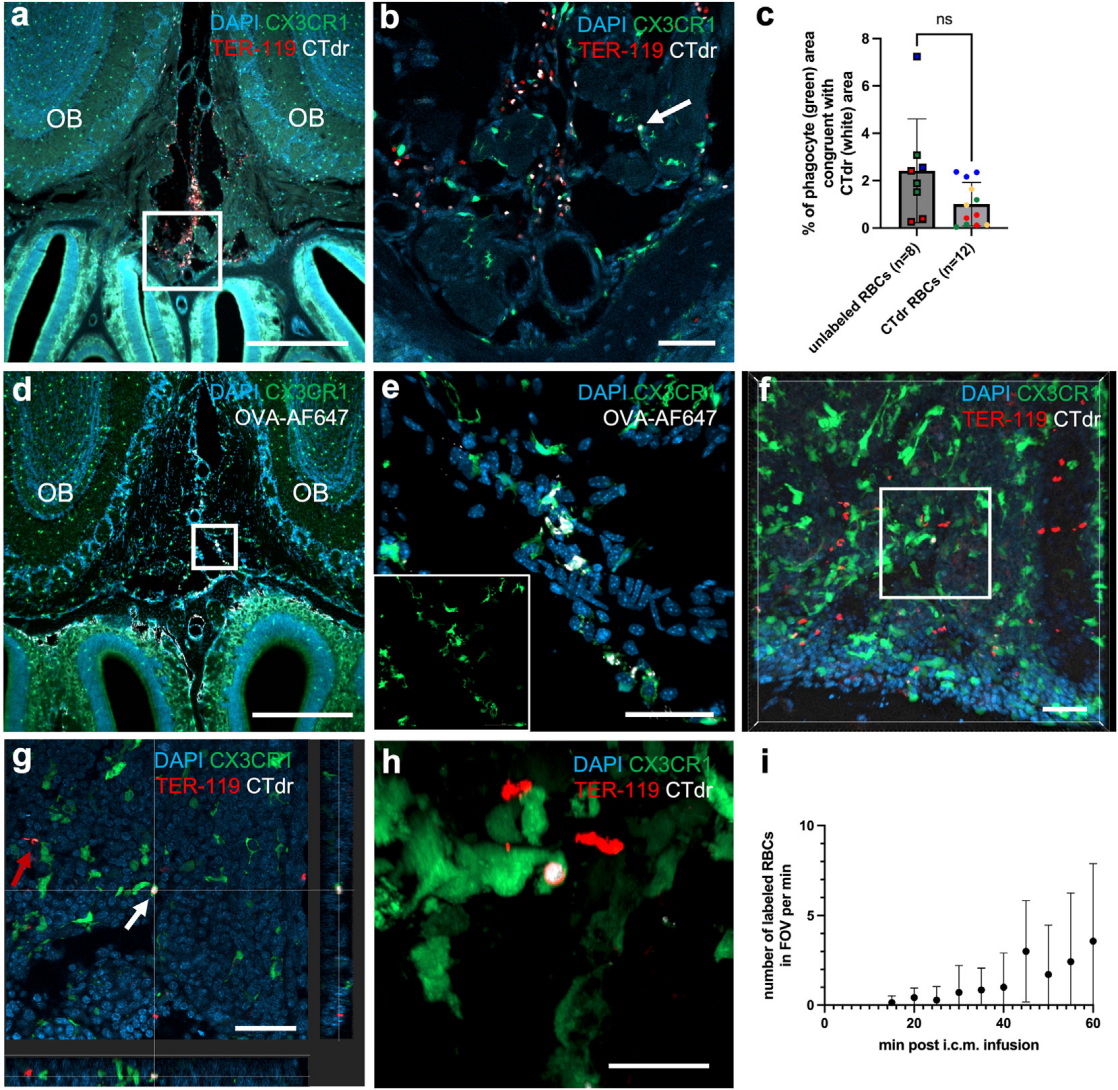
**Retention of RBCs in cervical lymph nodes but no evidence of phagocytosis in the SAS**. Representative coronal images of decalcified tissue at the cribriform plate region 2 h post-i.c.m. infusion of CTdr labelled autologous RBCs (n = 4). a) Overview showing distribution of CTdr-labelled RBCs (white = labelling, red = staining) along the midline between the olfactory bulbs. b) Magnification of a (white square). Only sporadic phagocytic (arrow) activity by resident phagocytes (green) of the CNS 2 h post i.c.m. infusion. c) No significant phagocytosis of labelled RBCs is evident within 2 h post-i.c.m. infusion (p = 0.0574, Mann–Whitney test two-tailed). Shown is the percentage of phagocyte (green) area congruent with CTdr (white) area. Same colour and shape dots mark data points acquired from one animal (3 in unlabelled, 4 in CTdr group). d) Representative coronal images of decalcified tissue at the cribriform plate region 2 h post-i.c.m. infusion of OVA-AF647 (n = 3). Overview. e) Magnification of d (white square), CX3XR1^+^ phagocytes (green) show OVA-AF647 uptake (white). Inlet: single channel. f) Representative image of dcLN 2 h post-i.c.m. infusion of CTdr-labelled RBCs in CX3CR1-GFP reporter mouse. Labelled RBCs drained from the SAS and are processed by phagocytes (n = 4). Intact RBCs as well as TER-119 stained RBC hulls without CTdr-labelled intracellular content are present. g) Close up orthogonal view of f (white square). Shown is a CTdr-labelled RBC being phagocytosed by a resident macrophage (centre, white arrow) as well as a morphologically no longer intact RBC likely undergoing lysis near a phagocyte (red arrow). h) 3D rendering of the RBC being phagocytosed shown in g. i) No significant increase of labelled RBCs crossing the FOV from 15 min up to 60 min post i.c.m. infusion (p = 0.0598, Kruskal–Wallis test with Dunn's correction, n = 7). Data presented as mean ± SD. ns = not significant. Scalebars: a,d 500 μm; b,e 50 μm; f,g, 40 μm; h 20 μm. OB = olfactory bulb.

In addition to efflux along CSF pathways, RBCs have been proposed to be cleared from the SAS by erythrophagocytosis.^46^^,^^47^^,^^57^^,^^58^ We investigated this mechanism 2 h post i.c.m. infusion of CTdr labelled RBCs using CX3CR1-GFP reporter mice to visualise CNS resident macrophages-mediated phagocytosis. Qualitative analysis of coronal cryosections at the cribriform plate region demonstrated only singular, sporadically occurring potential phagocytotic events (Fig. 5a and b). Some organelles within the CX3CR1-GFP^+^ phagocytes showed autofluorescence at the far-red fluorescence wavelengths rendering quantitative analysis of CTdr positivity difficult. This was overcome by staining the RBCs additionally for TER-119 and by comparing the CTdr signal in phagocytes in mice infused with labelled RBCs to mice infused with unlabelled RBCs. Statistical analysis showed no difference in far-red fluorescent area in phagocytes between mice infused with labelled vs. unlabelled RBCs (1.01 ± 0.92% vs. 2.42 ± 2.19%, p = 0.0574, Mann-whitney test two-tailed, 1 outlier removed in unlabelled RBC group due to elevated tissue autofluorescence), thus indicating no detectable phagocytosis of CTdr-labelled RBCs (Fig. 5c). To confirm the phagocytotic ability of CX3CR1-GFP^+^ cells near the cribriform plate we assessed OVA-AF647 uptake 2 h after i.c.m. infusion. Abundant OVA-AF647 uptake was apparent within CX3CR1-GFP^+^ phagocytes at the cribriform plate (Fig. 5d,e, Supplementary Fig. S8).

In contrast to the lack of erythrophagocytosis at the level of the cribriform plate, processing of RBCs in lymph nodes has been suggested to initiate rapidly, with RBCs being lysed or phagocytosed within 1 h.^47^^,^^76^ Confocal imaging of dcLNs harvested 2 h after i.c.m. infusion showed RBCs undergoing lysis visible by an empty TER-119 stained cell hull (Fig. 5f and g). Occasionally, RBCs engulfed by phagocytes could be visualised (Fig. 5g and h). This surprisingly high rate of retained and destructed RBCs prompted us to investigate if RBCs were able to drain from the lymph nodes back into the systemic circulation. Intermittent *in vivo* imaging of the microvasculature of the ear for 1 min and up to 60 min after i.c.m. infusion showed only single RBCs appeared to reach the systemic circulation (Supplementary Video S5). While at 60 min, single labelled RBCs could be detected in the bloodstream of all 7 animals investigated the number of cells detected did not significantly increase within 60 min unlike what is observed with inert tracers such as PEGylated dyes^38^ (Figs. 5i) (15 min 0.13 ± 0.38 vs 60 min 3.57 ± 4.32, p = 0.0598, Kruskal–Wallis test with Dunn's correction).

These findings, which demonstrate an undetectable erythrophagocytosis activity at the cribriform plate after 2 h but evidence of an efficient RBC processing in the dcLNs, further support the concept of a bulk flow efflux through the lymphatic system as the main mechanism for early RBC clearance from the SAS.

## Discussion

SAH primarily results from the rupture of cerebral aneurysms.^1^ Blood released into the SAS causes a sudden increase in ICP as well as severe impairment of CSF homeostasis.^8^, ^9^, ^10^, ^11^^,^^13^^,^^77^ Increases in ICP and the presence of free degradation products from RBC lysis contribute to EBI as well as DCI.^5^^,^^7^^,^^8^^,^^11^^,^^14^^,^^17^^,^^77^, ^78^, ^79^ Whilst the mechanisms governing clearance of RBCs from the SAS have attracted more interest lately, they remain incompletely understood. While studies using whole blood better represent the real situation of SAH, the analysis of RBC drainage pathways is complicated due to the presence of coagulating factors. Knowing if RBCs have the potential to be quickly cleared from the SAS can provide insightful information for developing SAH treatment strategies. Therefore, we aimed to elucidate the different clearance mechanisms and pathways for washed RBCs from the SAS. We demonstrated, employing i.c.m. infusions of labelled RBCs and P40D800 tracer followed by *in situ* fluorescence microscopy and histological analysis of potential efflux pathways from the SAS, that RBCs drain within 15 min through lymphatics at the cribriform plate to reach dcLNs. At the same time, we could detect only occasional instances of RBC entry into the dorsal dural lymphatics on either cryosections or dural whole mounts. Moreover, we found that RBCs draining to the dcLNs appear to be retained and lysed or phagocytosed within hours and thus only reach the systemic circulation in very limited numbers.

The surprisingly fast nature of RBC drainage, with cells arriving simultaneously with the P40D800 tracer, is indicative of a bulk outflow mechanism und thus implies open pathways patent for particles up to the size of 6–7 μm.^80^ Post-mortem imaging of the ventral side of the brain and the skull base revealed RBC and tracer spread along the ventral cisterns of the brain towards the cribriform plate area and along the optic nerves, confirming pathways already established for macromolecular tracer in previous investigations.^38^^,^^50^^,^^51^ Numerous studies have already shown the major contribution of lymphatics at the cribriform plate to CSF drainage to dcLNs in mice.^38^^,^^39^^,^^72^ Moreover, these pathways have been shown to be open for PEGylated beads of 1 μm size.^53^ Our high-resolution confocal images on decalcified tissue sections show RBCs accessing lymphatic vessels on the intracranial side of the cribriform plate. 3D reconstructions of this region illustrate how RBCs, once in the lymphatics, cross the cribriform plate within these vessels to reach the nasal submucosal lymphatics. Drainage to the lymphatics of the nasal mucosa is in line with previous studies that could find RBCs around olfactory nerve bundles or RBC breakdown products in the nasal mucosa or the dcLNs.^51^^,^^81^^,^^82^

Intriguingly, our data also demonstrates RBC efflux through lymphatics draining the orbital cavity 15–30 min after i.c.m. infusion. RBCs spreading along the perineural space of the optic nerve have been shown to reach the bulbar end, but there is no consensus as to how RBCs might overcome the arachnoid barrier at this location.^50^^,^^52^ Our investigations using E-cadherin immunofluorescence are in support of a continuous intact arachnoid membrane along the length of the optic nerve. However, we are still limited by the resolution of confocal imaging. Post-mortem investigations in patients that had succumbed to SAH showed parts of lysed RBCs, but not intact RBCs outside of the arachnoid barrier.^50^ Ultrastructural studies in cats could find evidence for a breakdown of the arachnoid barrier along the optic nerve following i.c.m. injection of autologous blood with an ICP peak equal to the arterial blood pressure.^52^ It is worth noting that these investigations were performed following SAH or by injecting substantial blood volumes into the SAS, both leading to a spike in ICP, possibly damaging the anatomical barriers. However, purely anatomical studies have demonstrated pore-like openings in the arachnoid at the end of the optic nerve.^83^ This concurs with reports of small particles leaking where the optic nerve enters the eye bulb.^50^^,^^84^ Whilst some RBCs might be washed off during immunofluorescence staining, the remaining RBCs are sufficiently abundant to perform our analyses as shown with the presence of RBCs in the optic nerve SAS. Proximal sections of the optic nerve in the region of the cavernous sinus showed RBCs outside of the E-cadherin^+^ positive arachnoid barrier layer. It remains to be elucidated if the RBCs can pass the arachnoid sheet of the optic nerve or if alternative pathways might allow for RBC efflux to the periorbital lymphatic plexus.

Following the rediscovery of dural lymphatics, many studies have postulated drainage of tracers and immune cells along these pathways.^40^^,^^41^^,^^75^ Our investigation of RBC drainage through dural lymphatics on decalcified cryosections as well as on dural whole mounts showed that drainage was significantly lower compared to the cribriform plate 30 min after i.c.m. infusion of RBCs. This is contradictory to previous findings in the literature as RBC efflux from the CNS through dorsal dural lymphatics was postulated in SAH, ICH and intraventricular haemorrhage models.^54^, ^55^, ^56^ For example, Chen et al. claimed that RBCs were draining through dorsal dural lymphatics in a whole-blood infusion model for SAH. However, in this study, large volumes of blood were injected, which raise concerns over the increase in ICP and the integrity of anatomical barriers. Another group reported RBC clearance through meningeal lymphatics in murine collagenase VII-S and blood infusion models of ICH.^55^ The results from these studies require careful interpretation as the images shown to support RBC drainage through meningeal lymphatics are epifluorescence images of dural whole mounts. As demonstrated in our work, it is impossible to determine the location of RBCs without 3D reconstruction of the whole mount tissue. Considering these results, our findings on the relative relevance of efflux pathways underline the importance of assessing all major routes. Further experiments are warranted to investigate the changes in the lymphatic vessels at the cribriform plate in intracranial haemorrhage models. However, despite considerable research efforts, histological evidence visually demonstrating that RBCs do not only sporadically access dural lymphatics intracranially but also drain along the dural lymphatic pathways proposed has yet to be provided. Furthermore, it remains elusive how RBCs can overcome the arachnoid barrier layer at the dorsal side of the brain.

In addition to efflux, erythrophagocytosis has been proposed as a mechanism for RBC removal from the SAS, particularly at later timepoints after stroke.^46^^,^^60^^,^^85^^,^^86^ The evidence supporting its involvement in the early removal of RBCs from the SAS remains inconclusive. In our experiments, no significant phagocytosis was evident at the cribriform plate 2 h post i.c.m. infusion of labelled RBCs in CX3CR1-GFP reporter mice. These results concur with the literature where erythrophagocytosis in the SAS after superficial intracerebral injection of washed autologous RBCs and microglia activation in a filament perforation model of SAH was only reported after 24 h.^47^^,^^60^ Previous investigations reported only non-phagocytosed RBCs draining to cervical lymph nodes, suggesting that the exit of phagocytes containing RBCs from the SAS does not significantly contribute to the clearance of RBCs. In contrast, the RBCs that reached the cervical lymph nodes were taken up by phagocytes within as little as 1 h following superficial intracerebral injection.^47^^,^^76^ We could confirm this finding, demonstrating lysis and erythrophagocytosis by CX3CR1 positive cells in the dcLNs by 2 h post i.c.m. infusion. Moreover, rapid activation of lysis and erythrophagocytosis could explain how only sporadic RBCs are visible in the systemic circulation 60 min after i.c.m. infusion even though many cells have reached the lymphatic vessels and the dcLNs by that timepoint.

Following SAH, also serum proteins and coagulant factors such as fibrin leak into the SAS and, together with RBC deposition, disturb CSF flow.^12^ In addition to the RBC clearance through cribriform plate lymphatics presented in this study, our group has shown that cribriform plate lymphatics are able to efficiently drain protein from the CSF spaces.^39^ The role of cribriform plate lymphatics in draining blood components, such as RBCs and proteins requires further investigation in murine SAH models. Due to the expected disruption of CSF circulation and efflux following SAH, intrathecal application of treatments is challenging, however, intraventricular application of antibodies to block astroglia-associated tissue factor has already been shown to improve CSF circulation in murine SAH models.^12^ Similar results were achieved by i.c.v. infusion of tissue plasminogen activator.^13^ Moreover, cribriform plate lymphatics have shown high plasticity adapting to changes in CNS homeostasis without pharmacological intervention in a model of neuroinflammation.^72^ However, the CNS and the CSF spaces are also accessible to drugs through minimally invasive intranasal delivery.^87^, ^88^, ^89^ In humans, the cribriform plate might contribute less to drainage due to a smaller relative size compared to rodents and RBC clearance might additionally occur through arachnoid granulations, as indicated by post-mortem studies.^19^^,^^49^^,^^90^Nonetheless, cribriform plate lymphatics present a target for future studies investigating RBC clearance from the SAS under pathological conditions.

The following limitations of this study should be noted. First, the resolution of confocal microscopy, while adequate for investigating RBC drainage pathways, does not provide the level of detail required for ultrastructural analysis of the arachnoid barrier. Second, rapid tracer movement in the SAS has been demonstrated upon death and a similar post-mortem artefact could not be excluded in this study.^91^ Additionally, the use of RBCs as tracers, valuable for mapping accessible pathways, did not fully replicate the complex composition of whole blood. Due to the diluted suspension of fluorescently labelled RBCs infused, we were able to investigate the pathways that are anatomically accessible for RBCs without confounding factors. However, this has limited our insight into the inflammatory effects and dynamic interactions of other blood components, such as clotting factors, within the SAS. Our findings should be interpreted in the context of these limitations, which may impact the generalisability of our results to the more complex pathophysiological cascade following SAH.

In summary, this study has investigated labelled RBC efflux from the SAS at different locations, providing evidence for a route of rapid RBC drainage through the cribriform plate and demonstrating the need for more research to elucidate the ultrastructure of the barriers around the optic nerve. Moreover, our data show, using 3D reconstructions of decalcified sections and dural whole mounts, that access of RBCs to the dural lymphatics at early timepoints after SAH is limited. With the insights gained in this study on the relevance and dynamics of different RBC clearance pathways, we emphasise the importance of investigating all major efflux pathways of the cranium. The data presented in this paper indicate that future research should examine the function of cribriform plate lymphatics in pathophysiological models of SAH.

## Contributors

A.M. and S.T.P conceived and designed the study, A.M. and L.X. performed *in vivo* experiments, A.M. and L.X. performed decalcified tissue analysis, A.M. and L.X. performed statistical analysis of data, A.M. and S.T.P drafted the manuscript. A.M. and S.T.P. have verified the underlying data and have the final responsibility for the decision to submit for publication. All the authors have read and approved the final version of the manuscript.

## Data sharing statement

The data supporting the findings of this study are available upon reasonable request to the corresponding author (steven.proulx@unibe.ch).

## Declaration of interests

A.M., L.X., and S.T.P. declare no conflict of interest.

## References

[1] Macdonald R.L., Schweizer T.A. (2017). Spontaneous subarachnoid haemorrhage. Lancet.

[2] Feigin V.L., Stark B.A., Johnson C.O. (2021). Global, regional, and national burden of stroke and its risk factors, 1990-2019: a systematic analysis for the Global Burden of Disease Study 2019. Lancet Neurol.

[3] Johnston S.C., Selvin S., Gress D.R. (1998). The burden, trends, and demographics of mortality from subarachnoid hemorrhage. Neurology.

[4] Darsaut T., Kotowski M., Raymond J. (2012). How to choose clipping versus coiling in treating intracranial aneurysms. Neurochirurgie.

[5] Neifert S.N., Chapman E.K., Martini M.L. (2021). Aneurysmal subarachnoid hemorrhage: the last decade. Transl Stroke Res.

[6] Kusaka G., Ishikawa M., Nanda A., Granger D.N., Zhang J.H. (2004). Signaling pathways for early brain injury after subarachnoid hemorrhage. J Cereb Blood Flow Metab.

[7] Gaasch M., Schiefecker A.J., Kofler M. (2018). Cerebral autoregulation in the prediction of delayed cerebral ischemia and clinical outcome in poor-grade aneurysmal subarachnoid hemorrhage patients. Crit Care Med.

[8] Dóczi T., Nemessányi Z., Szegváry Z., Huszka E. (1983). Disturbances of cerebrospinal fluid circulation during the acute stage of subarachnoid hemorrhage. Neurosurgery.

[9] Klimo P., Kestle J.R., MacDonald J.D., Schmidt R.H. (2004). Marked reduction of cerebral vasospasm with lumbar drainage of cerebrospinal fluid after subarachnoid hemorrhage. J Neurosurg.

[10] Milhorat T.H. (1987). Acute hydrocephalus after aneurysmal subarachnoid hemorrhage. Neurosurgery.

[11] van Gijn J., Hijdra A., Wijdicks E.F., Vermeulen M., van Crevel H. (1985). Acute hydrocephalus after aneurysmal subarachnoid hemorrhage. J Neurosurg.

[12] Golanov E.V., Bovshik E.I., Wong K.K. (2018). Subarachnoid hemorrhage - induced block of cerebrospinal fluid flow: role of brain coagulation factor III (tissue factor). J Cereb Blood Flow Metab.

[13] Siler D.A., Gonzalez J.A., Wang R.K., Cetas J.S., Alkayed N.J. (2014). Intracisternal administration of tissue plasminogen activator improves cerebrospinal fluid flow and cortical perfusion after subarachnoid hemorrhage in mice. Transl Stroke Res.

[14] Bulters D., Gaastra B., Zolnourian A. (2018). Haemoglobin scavenging in intracranial bleeding: biology and clinical implications. Nat Rev Neurol.

[15] Fujii M., Yan J., Rolland W.B., Soejima Y., Caner B., Zhang J.H. (2013). Early brain injury, an evolving frontier in subarachnoid hemorrhage research. Transl Stroke Res.

[16] Rass V., Helbok R. (2019). Early brain injury after poor-grade subarachnoid hemorrhage. Curr Neurol Neurosci Rep.

[17] Pradilla G., Chaichana K.L., Hoang S., Huang J., Tamargo R.J. (2010). Inflammation and cerebral vasospasm after subarachnoid hemorrhage. Neurosurg Clin.

[18] Engelhardt B., Vajkoczy P., Weller R.O. (2017). The movers and shapers in immune privilege of the CNS. Nat Immunol.

[19] Proulx S.T. (2021). Cerebrospinal fluid outflow: a review of the historical and contemporary evidence for arachnoid villi, perineural routes, and dural lymphatics. Cell Mol Life Sci.

[20] Sun B.L., Xie F.M., Yang M.F. (2011). Blocking cerebral lymphatic drainage deteriorates cerebral oxidative injury in rats with subarachnoid hemorrhage. Acta Neurochir Suppl.

[21] Louveau A., Plog B.A., Antila S., Alitalo K., Nedergaard M., Kipnis J. (2017). Understanding the functions and relationships of the glymphatic system and meningeal lymphatics. J Clin Invest.

[22] MacAulay N., Keep R.F., Zeuthen T. (2022). Cerebrospinal fluid production by the choroid plexus: a century of barrier research revisited. Fluids Barriers CNS.

[23] Mapunda J.A., Pareja J., Vladymyrov M. (2023). VE-cadherin in arachnoid and pia mater cells serves as a suitable landmark for in vivo imaging of CNS immune surveillance and inflammation. Nat Commun.

[24] Alcolado R., Weller R.O., Parrish E.P., Garrod D. (1988). The cranial arachnoid and pia mater in man: anatomical and ultrastructural observations. Neuropathol Appl Neurobiol.

[25] Nabeshima S., Reese T.S., Landis D.M., Brightman M.W. (1975). Junctions in the meninges and marginal glia. J Comp Neurol.

[26] Pietilä R., Del Gaudio F., He L. (2023). Molecular anatomy of adult mouse leptomeninges. Neuron.

[27] Balin B.J., Broadwell R.D., Salcman M., El-Kalliny M. (1986). Avenues for entry of peripherally administered protein to the central nervous system in mouse, rat, and squirrel monkey. J Comp Neurol.

[28] Yasuda K., Cline C., Vogel P. (2013). Drug transporters on arachnoid barrier cells contribute to the blood–cerebrospinal fluid barrier. Drug Metabol Dispos.

[29] Proulx S.T., Engelhardt B. (2022). Central nervous system zoning: how brain barriers establish subdivisions for CNS immune privilege and immune surveillance. J Intern Med.

[30] Key A., Retzius G. (1875).

[31] Weed L.H. (1914). Studies on cerebro-spinal fluid. No. III: the pathways of escape from the subarachnoid spaces with particular reference to the arachnoid villi. J Med Res.

[32] Cushing H. (1902). Some experimental and clinical observations concerning states of increased intracranial Tension.1: the mutter lecture for 1901. Am J Med Sci.

[33] Koh L., Zakharov A., Johnston M. (2005). Integration of the subarachnoid space and lymphatics: is it time to embrace a new concept of cerebrospinal fluid absorption?. Cerebrospinal Fluid Res.

[34] Bradbury M.W., Cserr H.F., Westrop R.J. (1981). Drainage of cerebral interstitial fluid into deep cervical lymph of the rabbit. Am J Physiol.

[35] Pollay M. (2010). The function and structure of the cerebrospinal fluid outflow system. Cerebrospinal Fluid Res.

[36] McComb J.G. (1983). Recent research into the nature of cerebrospinal fluid formation and absorption. J Neurosurg.

[37] Cserr H.F., Harling-Berg C.J., Knopf P.M. (1992). Drainage of brain extracellular fluid into blood and deep cervical lymph and its immunological significance. Brain Pathol.

[38] Ma Q., Ineichen B.V., Detmar M., Proulx S.T. (2017). Outflow of cerebrospinal fluid is predominantly through lymphatic vessels and is reduced in aged mice. Nat Commun.

[39] Decker Y., Krämer J., Xin L. (2022). Magnetic resonance imaging of cerebrospinal fluid outflow after low-rate lateral ventricle infusion in mice. JCI Insight.

[40] Louveau A., Smirnov I., Keyes T.J. (2015). Structural and functional features of central nervous system lymphatic vessels. Nature.

[41] Aspelund A., Antila S., Proulx S.T. (2015). A dural lymphatic vascular system that drains brain interstitial fluid and macromolecules. J Exp Med.

[42] Schwalbe G. (1869). Der Arachnoidalraum ein Lymphraum und sein Zusammenhang mit dem Perichorioidalraum. Zentralblatt fur die medizinischen Wissenschaften.

[43] Jacob L., de Brito Neto J., Lenck S. (2022). Conserved meningeal lymphatic drainage circuits in mice and humans. J Exp Med.

[44] Ahn J.H., Cho H., Kim J.-H. (2019). Meningeal lymphatic vessels at the skull base drain cerebrospinal fluid. Nature.

[45] Da Mesquita S., Louveau A., Vaccari A. (2018). Functional aspects of meningeal lymphatics in ageing and Alzheimer's disease. Nature.

[46] Xia F., Keep R.F., Ye F. (2022). The fate of erythrocytes after cerebral hemorrhage. Transl Stroke Res.

[47] Oehmichen M., Wietholter H., Gruninger H., Gencic M. (1983). Destruction of intracerebrally applied red blood cells in cervical lymph nodes. Experimental investigations. Forensic Sci Int.

[48] Oehmichen M., Gruninger H., Wietholter H., Gencic M. (1979). Lymphatic efflux of intracerebrally injected cells. Acta Neuropathol.

[49] Winkelman N.W., Fay T. (1930). The pacchionian system: histologic and pathologic changes with particular reference to the idiopathic and symptomatic convulsive states. Arch Neurol Psychiatr.

[50] Csanda E., Obal F., Obál F., Földi M., Casley-Smith J. (1983). Lymphangiology.

[51] Löwhagen P., Johansson B., Nordborg C. (1994). The nasal route of cerebrospinal fluid drainage in man. A light–microscope study. Neuropathol Appl Neurobiol.

[52] Brinker T., Ludemann W., von Rautenfeld D.B., Brassel F., Becker H., Samii M. (1997). Breakdown of the meningeal barrier surrounding the intraorbital optic nerve after experimental subarachnoid hemorrhage. Am J Ophthalmol.

[53] Spera I., Cousin N., Ries M. (2023). Open pathways for cerebrospinal fluid outflow at the cribriform plate along the olfactory nerves. eBioMedicine.

[54] Chen J., Wang L., Xu H. (2020). Meningeal lymphatics clear erythrocytes that arise from subarachnoid hemorrhage. Nat Commun.

[55] Tsai H.-H., Hsieh Y.-C., Lin J.S. (2022). Functional investigation of meningeal lymphatic system in experimental intracerebral hemorrhage. Stroke.

[56] Li D., Liu S., Yu T. (2023). Photostimulation of brain lymphatics in male newborn and adult rodents for therapy of intraventricular hemorrhage. Nat Commun.

[57] Adams J.E., Prawirohardjo S. (1959). Fate of red blood cells injected into cerebrospinal fluid pathways. Neurology.

[58] Carpenter S.J., McCarthy L.E., Borison H.L. (1967). Morphologic and functional effects of intracerebroventricular administration of autologous blood in cats. Neurology.

[59] Dupont J.-R., Wart C.A.V., Kraintz L. (1961). The clearance of major components of whole blood from cerebrospinal fluid following simulated subarachnoid hemorrhage. J Neuropathol Exp Neurol.

[60] Xu Z., Shi W.H., Xu L.B. (2019). Resident microglia activate before peripheral monocyte infiltration and p75NTR blockade reduces microglial activation and early brain injury after subarachnoid hemorrhage. ACS Chem Neurosci.

[61] Choi I., Chung H.K., Ramu S. (2011). Visualization of lymphatic vessels by Prox 1-promoter directed GFP reporter in a bacterial artificial chromosome-based transgenic mouse. Blood.

[62] Saederup N., Cardona A.E., Croft K. (2010). Selective chemokine receptor usage by central nervous system myeloid cells in CCR2-red fluorescent protein knock-in mice. PLoS One.

[63] Fukuda T., Asou E., Nogi K., Goto K. (2017). Evaluation of mouse red blood cell and platelet counting with an automated hematology analyzer. J Vet Med Sci.

[64] Albargothy N.J., Johnston D.A., MacGregor-Sharp M. (2018). Convective influx/glymphatic system: tracers injected into the CSF enter and leave the brain along separate periarterial basement membrane pathways. Acta Neuropathol.

[65] Proulx S.T., Luciani P., Christiansen A. (2013). Use of a PEG-conjugated bright near-infrared dye for functional imaging of rerouting of tumor lymphatic drainage after sentinel lymph node metastasis. Biomaterials.

[66] Smith A.J., Akdemir G., Wadhwa M., Song D., Verkman A.S. (2021). Application of fluorescent dextrans to the brain surface under constant pressure reveals AQP4-independent solute uptake. J Gen Physiol.

[67] Schindelin J., Arganda-Carreras I., Frise E. (2012). Fiji: an open-source platform for biological-image analysis. Nat Methods.

[68] Louveau A., Filiano A.J., Kipnis J. (2018). Meningeal whole mount preparation and characterization of neural cells by flow cytometry. Curr Protoc Im.

[69] Ma Q., Schlegel F., Bachmann S.B. (2019). Lymphatic outflow of cerebrospinal fluid is reduced in glioma. Sci Rep.

[70] Yoon J.H., Jin H., Kim H.J. (2024). Nasopharyngeal lymphatic plexus is a hub for cerebrospinal fluid drainage. Nature.

[71] Ma Q., Decker Y., Muller A., Ineichen B.V., Proulx S.T. (2019). Clearance of cerebrospinal fluid from the sacral spine through lymphatic vessels. J Exp Med.

[72] Hsu M., Rayasam A., Kijak J.A. (2019). Neuroinflammation-induced lymphangiogenesis near the cribriform plate contributes to drainage of CNS-derived antigens and immune cells. Nat Commun.

[73] Killer H.E., Laeng H.R., Groscurth P. (1999). Lymphatic capillaries in the meninges of the human optic nerve. J Neuro Ophthalmol.

[74] Shen J.Y., Kelly D.E., Hyman S., McComb J.G. (1985). Intraorbital cerebrospinal fluid outflow and the posterior uveal compartment of the hamster eye. Cell Tissue Res.

[75] Louveau A., Herz J., Alme M.N. (2018). CNS lymphatic drainage and neuroinflammation are regulated by meningeal lymphatic vasculature. Nat Neurosci.

[76] Oehmichen M., Wiethölter H., Wolburg H. (1982). Enhanced phagocytic activity of lymph node macrophages after intranodular injection of autologous red blood cells. Z Rechtsmed.

[77] Grote E., Hassler W. (1988). The critical first minutes after subarachnoid hemorrhage. Neurosurgery.

[78] Sehba F.A., Pluta R.M., Zhang J.H. (2011). Metamorphosis of subarachnoid hemorrhage research: from delayed vasospasm to early brain injury. Mol Neurobiol.

[79] Weir B., Macdonald R.L., Stoodley M. (1999). Etiology of cerebral vasospasm. Acta Neurochir Suppl.

[80] Namdee K., Carrasco-Teja M., Fish M.B., Charoenphol P., Eniola-Adefeso O. (2015). Effect of variation in hemorheology between human and animal blood on the binding efficacy of vascular-targeted carriers. Sci Rep.

[81] Caversaccio M., Peschel O., Arnold W. (1996). The drainage of cerebrospinal fluid into the lymphatic system of the neck in humans. ORL J Otorhinolaryngol Relat Spec.

[82] García-Cabo C., Llano-Suárez P., Benavente-Fernández L., Calleja-Puerta S., Costa-Fernández J.M., Fernández-Abedul M.T. (2020). Obtaining information from the brain in a non-invasive way: determination of iron in nasal exudate to differentiate hemorrhagic and ischemic strokes. Clin Chem Lab Med.

[83] Ludemann W., von Rautenfeld D.B., Samii M., Brinker T. (2005). Ultrastructure of the cerebrospinal fluid outflow along the optic nerve into the lymphatic system. Child's Nerv Syst.

[84] Field E.J., Brierley J.B. (1949). The retro-orbital tissues as a site of outflow of cerebrospinal fluid. Proc Roy Soc Med.

[85] Kwon S., Janssen C.F., Velasquez F.C., Sevick-Muraca E.M. (2017). Fluorescence imaging of lymphatic outflow of cerebrospinal fluid in mice. J Immunol Methods.

[86] Wan H., Brathwaite S., Ai J., Hynynen K., Macdonald R.L. (2021). Role of perivascular and meningeal macrophages in outcome following experimental subarachnoid hemorrhage. J Cereb Blood Flow Metab.

[87] Pernet V., Joly S., Spiegel S. (2023). Nogo-A antibody delivery through the olfactory mucosa mitigates experimental autoimmune encephalomyelitis in the mouse CNS. Cell Death Discov.

[88] Lochhead J.J., Kumar N.N., Nehra G., Stenslik M.J., Bradley L.H., Thorne R.G. (2022).

[89] Correa D., Scheuber M.I., Shan H. (2023). Intranasal delivery of full-length anti-Nogo-A antibody: a potential alternative route for therapeutic antibodies to central nervous system targets. Proc Natl Acad Sci USA.

[90] Ellington E., Margolis G. (1969). Block of arachnoid villus by subarachnoid hemorrhage. J Neurosurg.

[91] Ma Q., Ries M., Decker Y. (2019). Rapid lymphatic efflux limits cerebrospinal fluid flow to the brain. Acta Neuropathol.

