# Supplementary material for: Clearance of erythrocytes from the subarachnoid space through cribriform plate lymphatics in female mice
Source: eBioMedicine. 2024 Aug 22;107:105295. doi: 10.1016/j.ebiom.2024.105295 (PMC11388277; doi:10.1016/j.ebiom.2024.105295)
Supplement: Supplementary Figures [file mmc1.pdf]

Supplementary material

Erythrocytes Clear from the Subarachnoid Space to Lymphatics Through the Cribriform Plate

Adrian Madarasz[1], Li Xin[1] and Steven T. Proulx[1]

[1] Theodor Kocher Institute, University of Bern, Bern, Switzerland

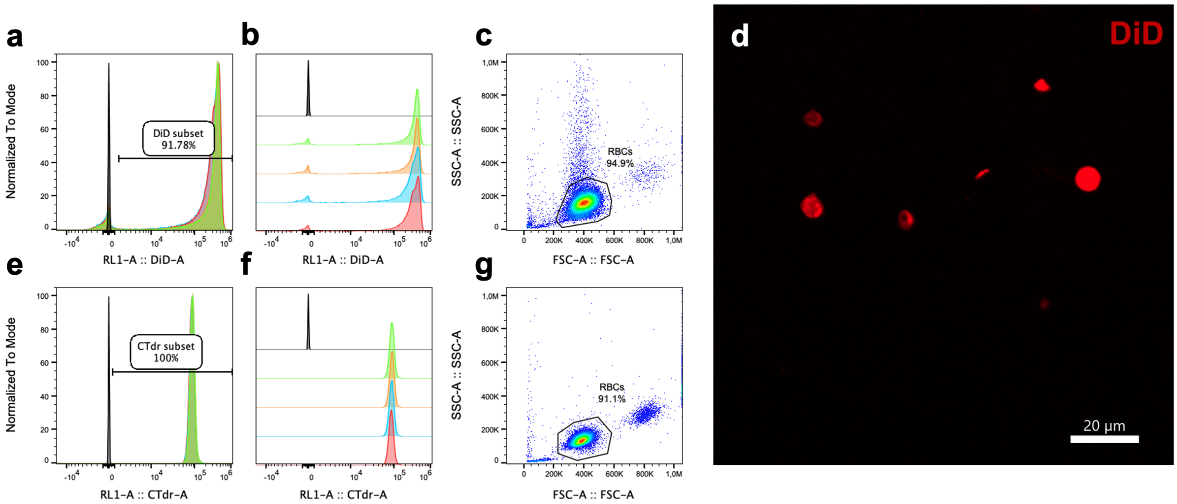

a
b
c
d
DiD
Normalized To Mode
DiD subset
91.78%
RL1-A :: DiD-A
RL1-A :: DiD-A
SSC-A :: SSC-A
RBCs
94.9%
FSC-A :: FSC-A
20 µm
e
f
g
CTdr subset
100%
RL1-A :: CTdr-A
RL1-A :: CTdr-A
RBCs
91.1%
FSC-A :: FSC-A

Supplementary Figure 1. RBC labelling validation

Histogram a) and half-offset histogram b) showing DiD labelled subset of RBCs directly after staining in 4 replicates. c) Representative gating strategy for DiD labelled RBCs. d) Representative confocal image showing morphologically intact single RBCs after DiD labelling. Histogram e) and half-offset histogram f) showing CTdr labelled subset of RBCs directly after staining in 4 replicates. g) Representative gating strategy for CTdr labelled RBCs.

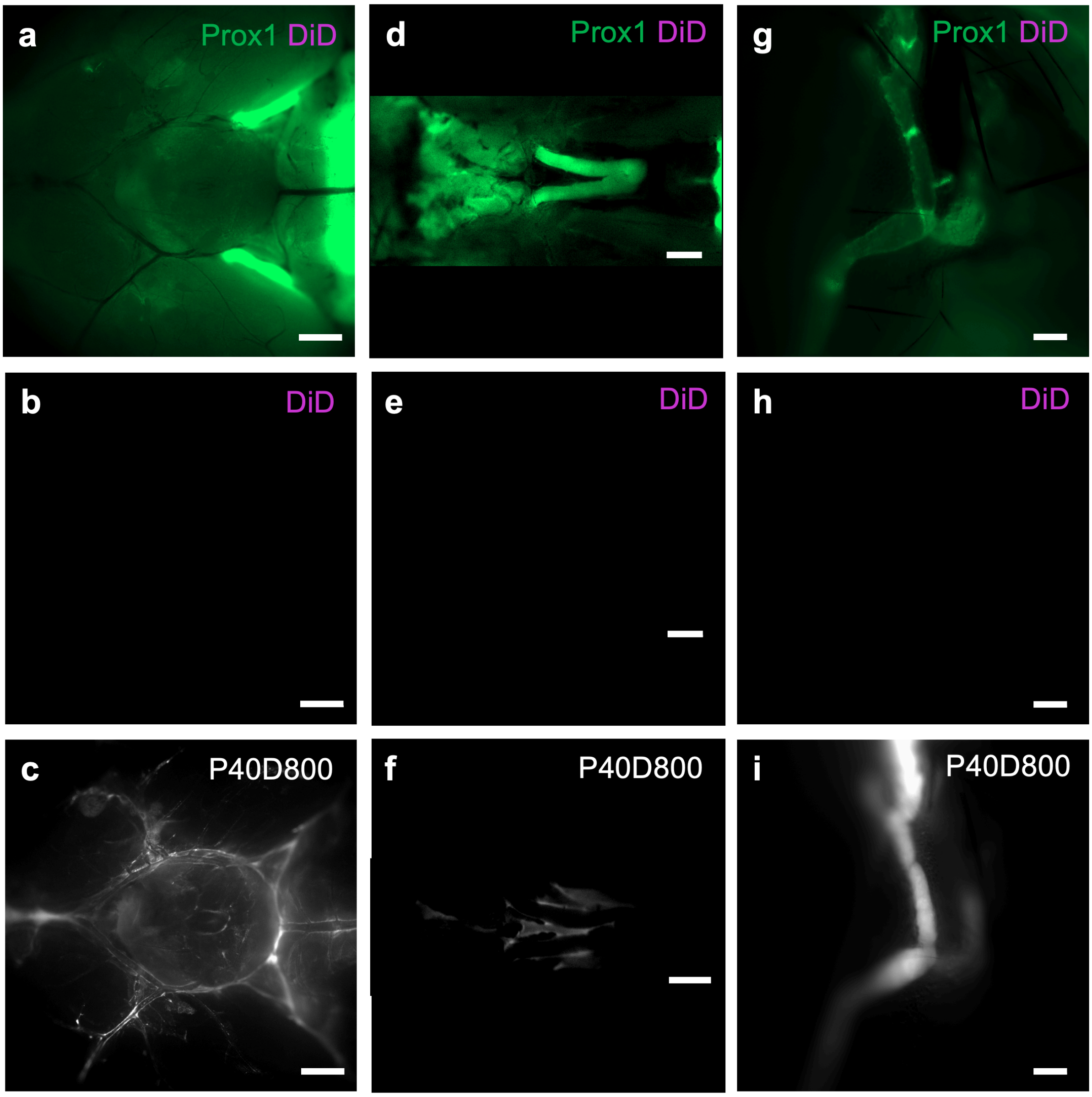
a
Prox1 DiD
d
Prox1 DiD
g
Prox1 DiD
b
DiD
e
DiD
h
DiD
c
P40D800
f
P40D800
i
P40D800

Supplementary Figure 2. Controls with i.c.m. infusion of unlabelled RBCs and near-infrared tracer

a,b,c) Representative image of the ventral cisterns of the brain 15 min post i.c.m. infusion of unlabelled RBCs (1.5µl, 1.5x10^6 RBCs) and P40D800 near-infrared tracer (1.5µl). d,e,f) Representative image of the skull base 15 min post i.c.m. infusion. g,h,i) Representative control image of the lymphatics (green) draining the orbit 15 min post i.c.m. infusion (n=3). Scalebars: *a,b,c,d,e,f 1000µm; g,h,i 200µm.*

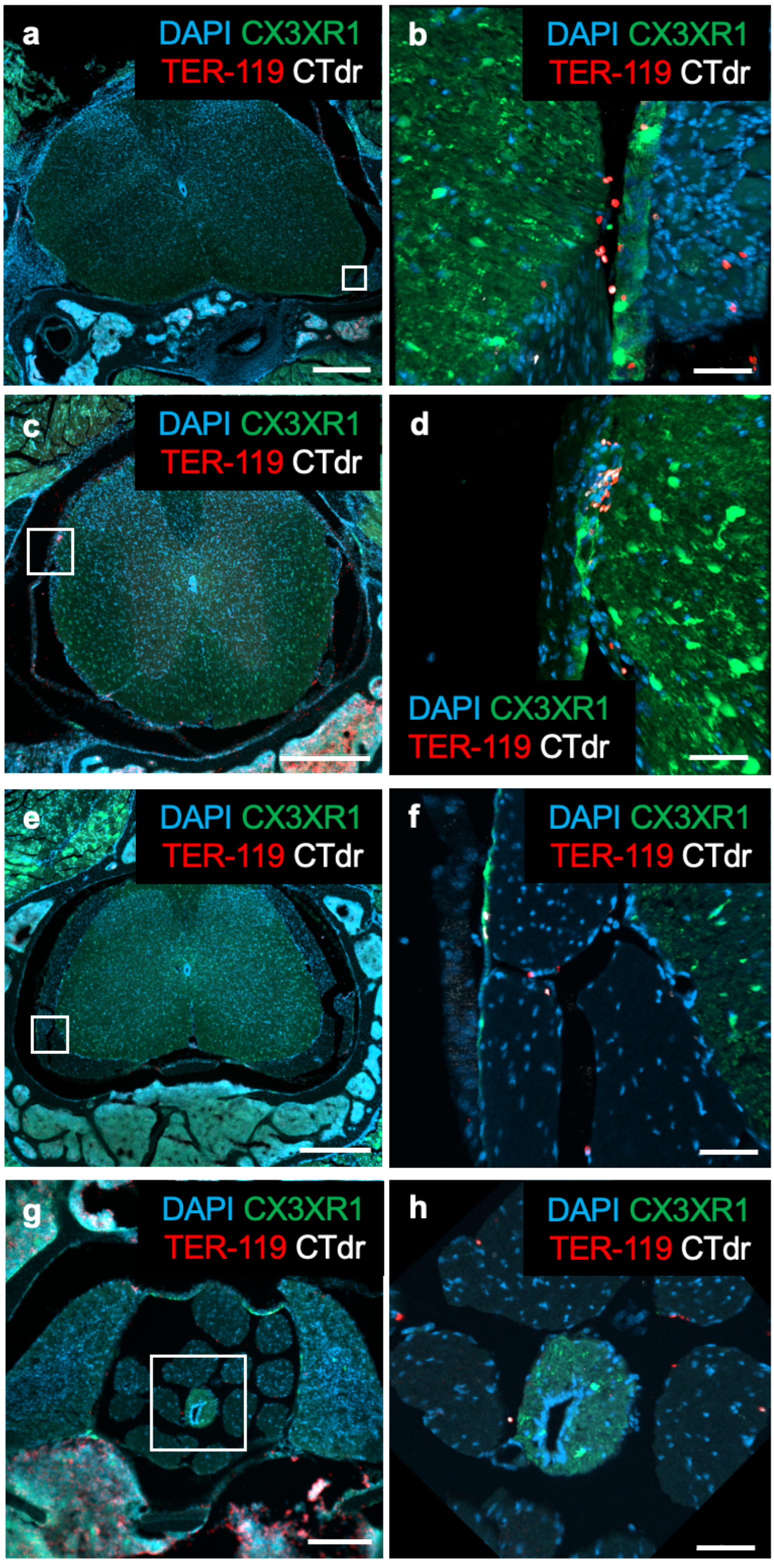
a
DAPI CX3XR1
TER-119 CTdr
b
DAPI CX3XR1
TER-119 CTdr
c
DAPI CX3XR1
TER-119 CTdr
d
DAPI CX3XR1
TER-119 CTdr
e
DAPI CX3XR1
TER-119 CTdr
f
DAPI CX3XR1
TER-119 CTdr
g
DAPI CX3XR1
TER-119 CTdr
h
DAPI CX3XR1
TER-119 CTdr

Supplementary Figure 3. Minor RBC flow along the spinal SAS 30 min after i.c.m. infusion

Representative images of the spine from decalcified sections 30 min post i.c.m. infusion of CTdr labelled autologous RBCs (n=4). a,b) cervical level and b,c) thoracic level show minor RBC accumulations (white = labelling, red = staining). e,f) lumbar and g,h) sacral levels of the spinal cord. Only singular labelled cells are detectable. Maximum intensity projections in b,d,f,h. *Scalebars: a,c,e 500µm; g 200µm; b,d,f,h 50µm.*

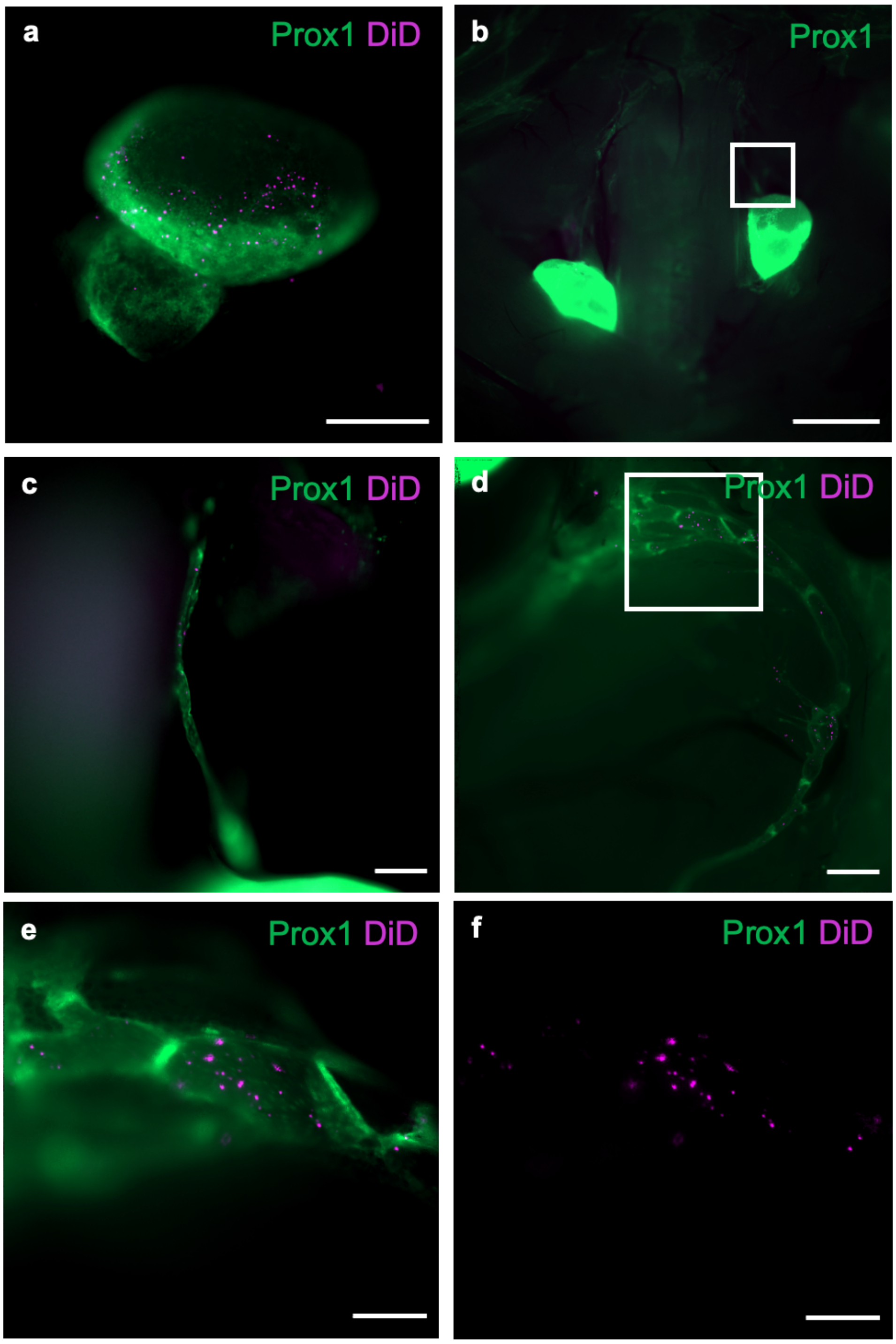
a
Prox1 DiD
b
Prox1
c
Prox1 DiD
d
Prox1 DiD
e
Prox1 DiD
f
Prox1 DiD

Supplementary Figure 4. Exemplary images of DiD labelled RBCs draining in lymphatic vessels following i.c.m. infusion at different timepoints

a) Representative extended depth of focus image of a dcLN (green) containing DiD labelled RBCs (pink) 30 min after i.c.m. infusion (n=7). b) Representative overview of exposed dcLNs 60 min after i.c.m. infusion of labelled RBCs (n=8). c) Magnification of b. RBCs are present in the afferent lymphatic vessel of the dcLN. d) Representative extended depth of focus image of lymphatics draining the eye 30 min after i.c.m. infusion. Eye in left upper corner (n=7). e) Magnification of e. Extended depth of focus image. f) Single channel image of e. *Scalebars: a,d 500µm; b 2000µm; c,e,f 200µm.*

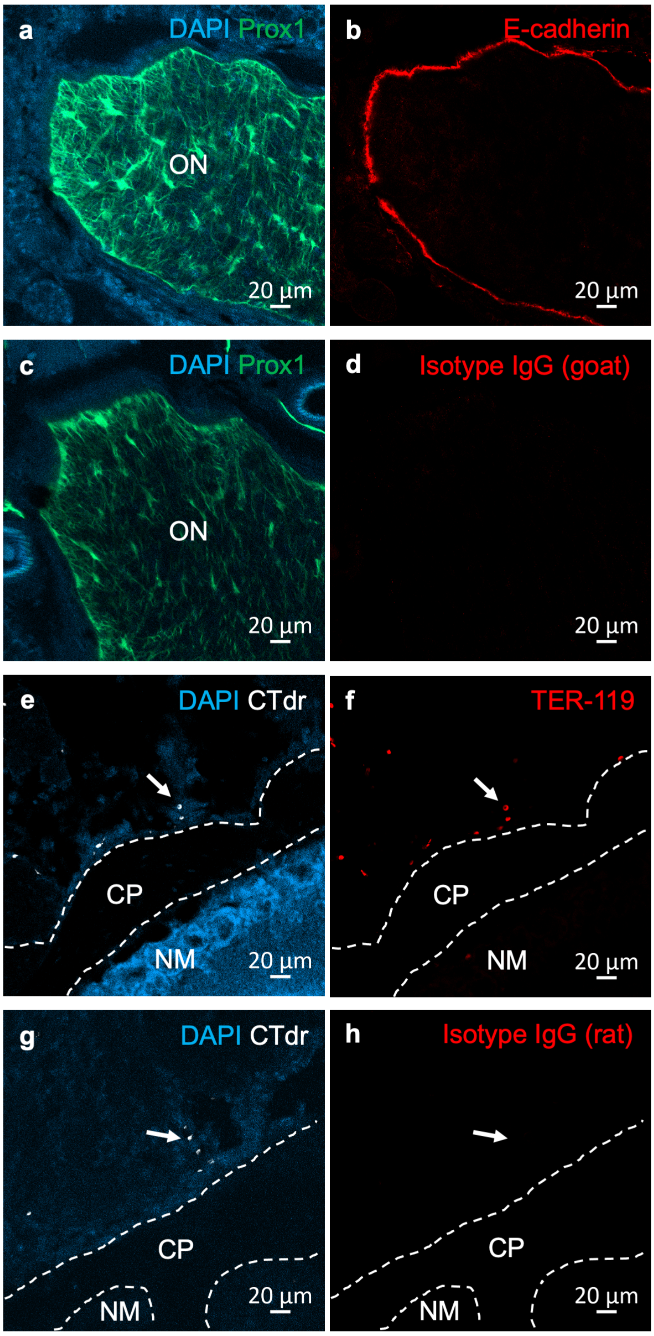
a
DAPI Prox1
ON
20 µm
b
E-cadherin
20 µm
c
DAPI Prox1
ON
20 µm
d
Isotype IgG (goat)
20 µm
e
DAPI CTdr
CP
NM
20 µm
f
TER-119
CP
NM
20 µm
g
DAPI CTdr
CP
NM
20 µm
h
Isotype IgG (rat)
CP
NM
20 µm

Supplementary Figure 5. Isotype controls for E-cadherin and TER-119 immunofluorescence staining

a-d) Representative images of the optic nerve from coronal decalcified sections in Prox1-EGFP reporter mice stained with DAPI and goat anti-E-cadherin antibody (b) or isotype IgG (d, n=3). e-h) Representative images of the cribriform plate from coronal decalcified sections in Prox1-EGFP reporter mice (channel not displayed) after i.c.m. infusion with CTdr labelled RBCs, stained with DAPI and rat anti-TER-119 antibody (f) or isotype IgG (h,n=3). *ON = optic nerve, CP = cribriform plate, NM = nasal mucosa*

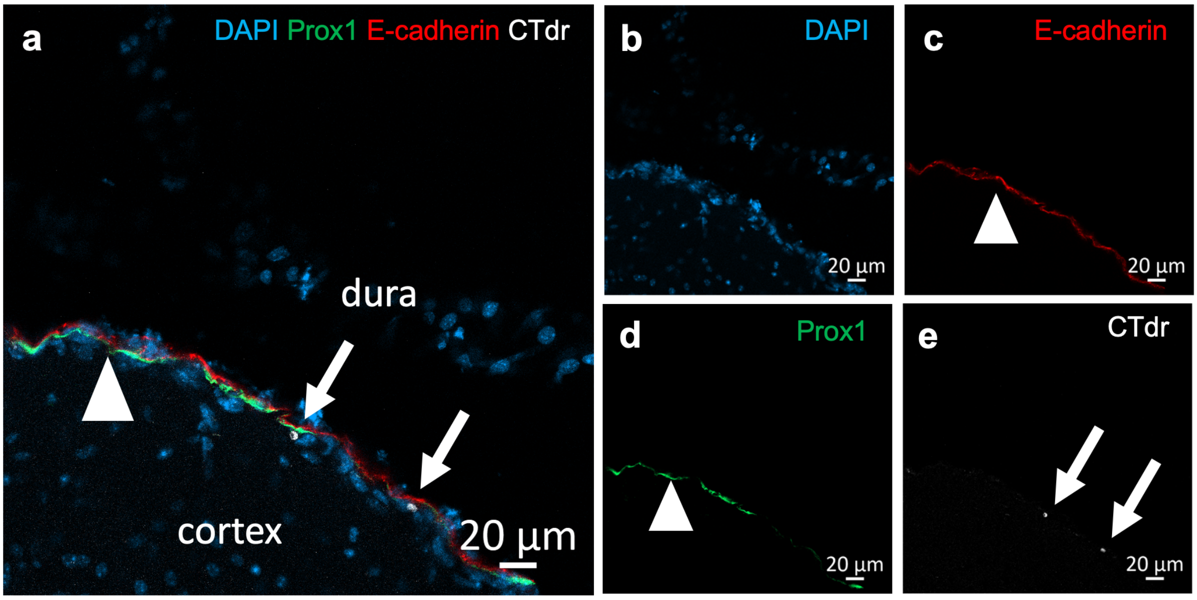

a
DAPI Prox1 E-cadherin CTdr
dura
cortex
20 µm
b
DAPI
20 µm
c
E-cadherin
20 µm
d
Prox1
20 µm
e
CTdr
20 µm

Supplementary Figure 6. RBCs can be localised in the SAS by using the E-cadherin staining and Prox1-EGFP reporter signal of the arachnoid as landmark
a) Representative sagittal image of decalcified tissue of the interface between cortex and meninges 30 min post i.c.m. infusion of CTdr labelled RBCs (1.5µl, 1.5x10^6 RBCs) and P40D800 near-infrared tracer (1.5µl). Image showing E-cadherin layer (red) is associated with Prox1-EGFP (green) signal along arachnoid mater. b,c,d,e) Single channel images of a. RBCs are located below the arachnoid mater in the subarachnoid space. arrowhead = arachnoid mater, arrows = CTdr labelled RBCs.

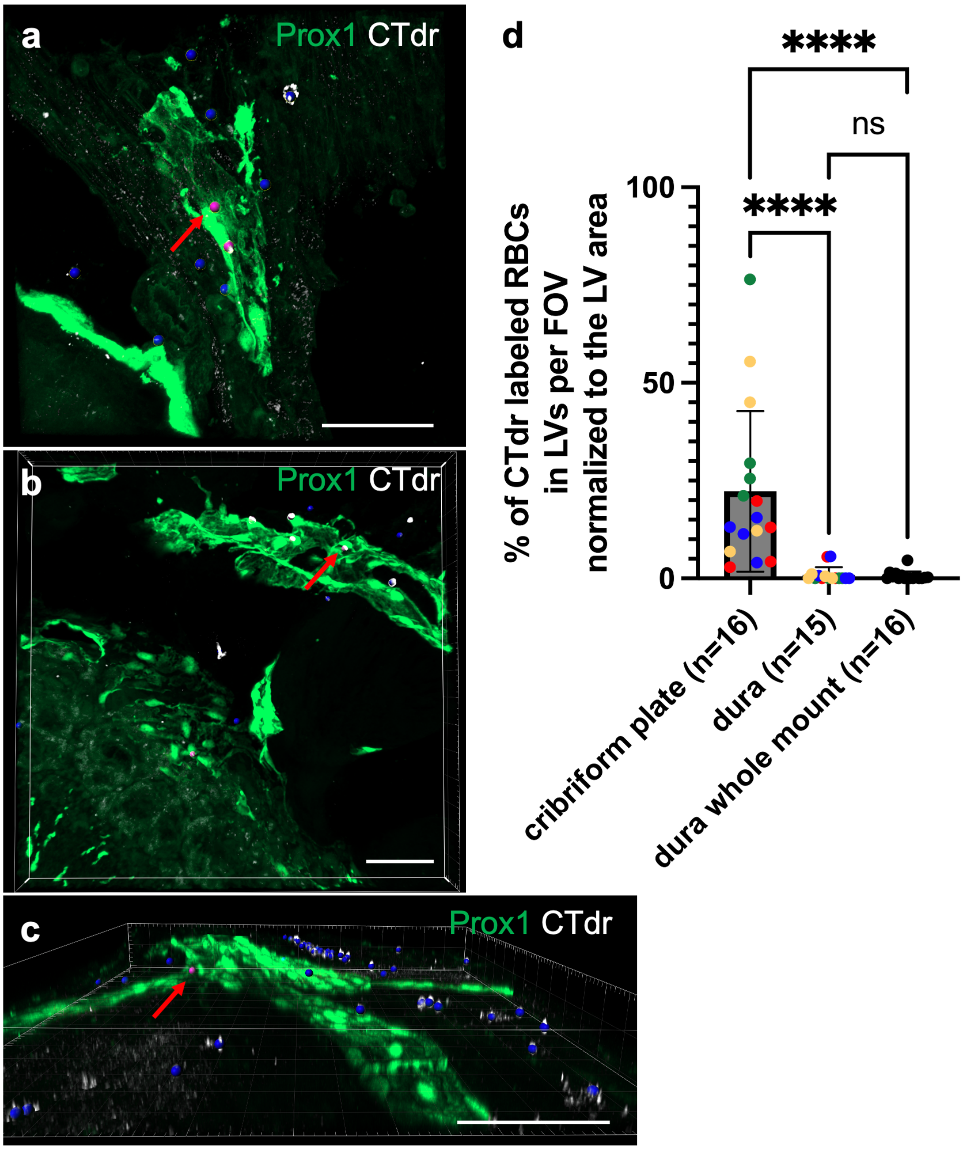
a
Prox1 CTdr
b
Prox1 CTdr
c
Prox1 CTdr
d
****
ns
****
% of CTdr labeled RBCs in LVs per FOV normalized to the LV area
100
50
0
cribriform plate (n=16)
dura (n=15)
dura whole mount (n=16)

Supplementary Figure 7. Quantification of RBCs in lymphatic vessels

Exemplary images to illustrate the quantification of RBCs in lymphatic vessels. RBCs were counted with Imaris spot detection function based on the CTdr channel and spot distance to the green channel depicting the lymphatics measured. Examples of spots considered to be associated with the green channels are marked with red arrows. All spots were controlled manually and corrected where needed. a) Representative sagittal 3D reconstruction of dorsal dural lymphatics from z-stacks acquired on decalcified tissue, 30 min post i.c.m. infusion. Imaging close to the confluence of sinus (n=4). b) Representative coronal 3D reconstruction of cribriform plate lymphatics from z-stacks acquired on decalcified tissue, 30 min post i.c.m. infusion (n=4). c) Representative 3D reconstruction of dorsal dural lymphatics from z-stacks acquired on a dura whole mount, 30 min post i.c.m. infusion (n=4). d) Quantification of RBCs inside the lymphatic vessel as percentage of all RBCs in the field of view normalised to the area occupied by the lymphatic vessels to adjust for different vessel calibers and structures at the locations imaged. Lymphatic vessel area was measured by uniform threshold application on maximum intensity projections. Same colour dots mark data points acquired from one animal. **** = $p < 0.0001$ (Kruskal-Wallis test with Dunn's correction). Data presented as mean ± SD. *Scalebars: a,b, 50µm; c 100µm.*

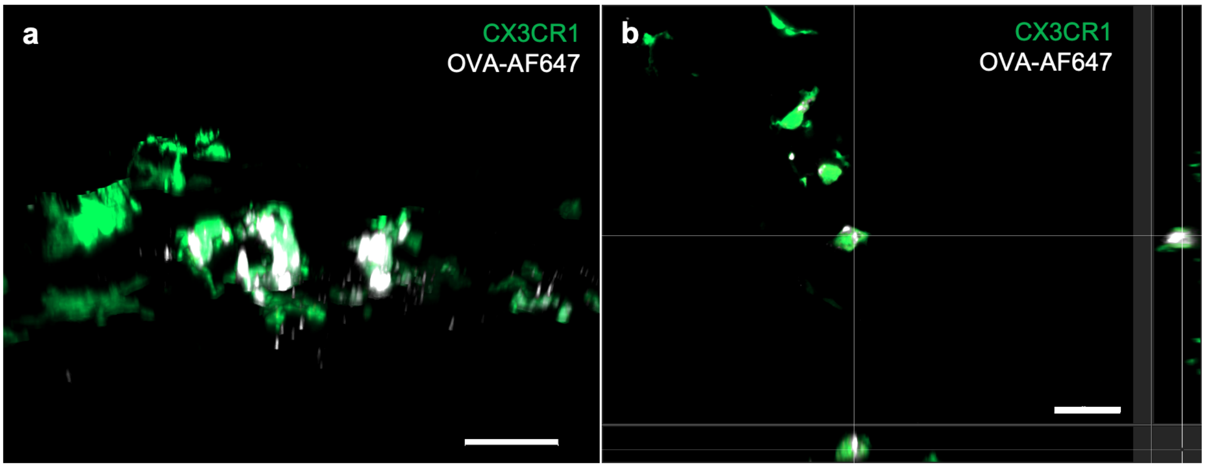
a
CX3CR1
OVA-AF647
b
CX3CR1
OVA-AF647

Supplementary Figure 8. OVA-AF647 is phagocytosed by $CX3CR1^{+}$ resident phagocytes at the cribriform plate within 2 hours

Representative images of OVA-AF647 (white) phagocytosis by $CX3CR1^{+}$ resident phagocytes (green) above the cribriform plate 2 h after i.c.m. infusion (n=3). Imaged location equivalent to fig. 5e, higher magnification. a) 3D reconstruction from confocal imaging. b) Orthogonal view demonstrating OVA-AF647 inside of the phagocyte. *Scalebars: a,b, 20μm.*

Supplementary Video 1. Intravenously injected DiD labelled RBCs provide strong signal

Representative *in vivo* imaging of autologous DiD labelled RBCs (pink) circulating in the ear's microvasculature directly after intravenous injection. Outline of the vessel in white. Vessel visible as dark streaks against the autofluorescence of the tissue and the EGFP signal (green) in the skin lymphatics.

Supplementary Video 2. RBCs in the SAS can access lymphatic vessels associated with olfactory nerve bundles at the cribriform plate

Representative z-stack of coronal images of decalcified tissue at the cribriform plate region 30 min post i.c.m. infusion of CTdr labelled autologous RBCs (1.5µl, 1.5x10^6 RBCs, white = label, red = TER-119 staining). RBCs can be seen within the lumen of lymphatic vessels (green) crossing the cribriform plate (blue = DAPI). Tissue and location identical to Fig. 3c.

Supplementary Video 3. RBCs clear from the SAS through lymphatic vessels of the nasal mucosa

Representative 3D reconstruction of a coronal image of decalcified tissue at the cribriform plate region 30 min post i.c.m. infusion of CTdr labelled autologous RBCs (1.5µl, 1.5x10^6 RBCs, white = label, red = staining). RBCs are shown clearing through the lymphatic vessels (green) of the nasal mucosa below the cribriform plate (blue = DAPI). Location identical to Fig. 3f.

Supplementary Video 4. 3D rendering demonstrating RBCs infused into the SAS are not associated with dorsal dural lymphatics

Video of a representative 3D reconstruction of a dural whole mount preparation harvested 30 min after i.c.m. infusion of CTdr labelled autologous RBCs (1.5µl, 1.5x10^6 RBCs) demonstrating labelled RBCs (white) mainly outside of dorsal dural lymphatics (green).

Supplementary Video 5. Labelled RBCs reaching systemic circulation following i.c.m. infusion

Representative *in vivo* imaging of autologous DiD labelled RBCs (pink) circulating in the ear's microvasculature 25 min after i.c.m. infusion. Outline of the vessel in white. Vessel visible as dark streaks against the autofluorescence of the tissue and the EGFP signal (green) in the skin lymphatics. *Scalebar 100µm.*
